# Biocontrol Mechanism of *Bacillus subtilis* C3 Against Bulb Rot Disease in *Fritillaria taipaiensis* P.Y.Li

**DOI:** 10.3389/fmicb.2021.756329

**Published:** 2021-09-30

**Authors:** Yongli Ku, Nan Yang, Peng Pu, Xueli Mei, Le Cao, Xiangna Yang, Cuiling Cao

**Affiliations:** ^1^College of Life Sciences, Northwest A&F University, Yangling, China; ^2^College of Chemistry and Pharmacy, Northwest A&F University, Yangling, China; ^3^College of Environment and Life Sciences, Weinan Normal University, Weinan, China

**Keywords:** *Fritillaria taipaiensis* P.Y.Li, *Bacillus subtilis*, *Fusarium*, active ingredients, biological control

## Abstract

Bulb rot disease has become one of the main diseases that seriously affects the yield and quality of *Fritillaria taipaiensis* P.Y.Li (*F. taipaiensis*). In this study, *F. taipaiensis* was used as the research object to explore the effect and mechanism of *Bacillus subtilis* C3 in preventing and curing bulb rot. Through isolation and verification of the pathogenic fungi, we determined for the first time that the pathogenic fungus that causes bulb rot in *F. taipaiensis* is *Fusarium oxysporum*. The results of the study showed that *B. subtilis* C3 inhibits the growth of pathogenic fungi, and the inhibition rate is as high as 60%. In the inhibition mechanism, strain C3 inhibits the conidiogenesis of pathogenic fungi and destroys the cell structure of its hyphae, causing protoplast exudation, chromatin concentration, DNA fragmentation, and ultimately cell death. Among the secondary metabolites of C3, antimicrobial proteins and main active components (paeonol, ethyl palmitate, and oxalic acid) inhibited the growth of *F. oxysporum*. The molecular weight of the antibacterial protein with the highest inhibition rate was approximately 50 kD. The results of a field experiment on the Taibai Mountain *F. taipaiensis* planting base showed that after the application of strain C3, the incidence of bulb rot in *Fritillaria* was reduced by 18.44%, and the ratio of bacteria to fungi in the soil increased to 8.21, which verified the control effect of C3 on *Fritillaria* bulb rot disease. This study provides a theoretical basis for the use of *B. subtilis* C3 to prevent and control bulb rot in *Fritillaria*.

## Introduction

Bulbus *Fritillaria* (i.e., dry bulbs of *Fritillaria* species; “Beimu” in Chinese) is derived from the bulbs of many *Fritillaria* species and has been used as one of the most important antitussive, expectorant, and antihypertensive drugs in traditional Chinese medicine (TCM) for up to 2000 years (Ge et al., 2001; Li et al., 2013). *Fritillaria taipaiensis* P.Y.Li (Taibai Beimu in Chinese) is one of the *Fritillaria* species used in TCM. It has a long planting history in Shaanxi Province and is considerably beneficial to the farmers who plant it. In recent years, the planting area of *Fritillaria* has gradually expanded, but *Fritillaria* disease has become increasingly serious, which has severely affected the development of the *Fritillaria* planting industry (Zuchen et al., 2021). Some articles have shown that the main diseases of *Fritillaria* are as follows: sclerotinia, which is caused by *Stromatinia rapulum* (Sun et al., 2019) and mainly destroys the bulb and stem base of *Fritillaria*; rust, the main causative pathogen of which is *Uromyces lilli*, which infects the stem and leaf, resulting in premature withering and death of the aboveground part of *Fritillaria* (Zhu and Zheng, 2008); andash, which is caused by *Botrytis elliptica* (Zong et al., 2018), that mainly infects leaves, stems, and flowers and leads to the wilting and death of plants.

Among plant diseases, 70–80% are caused by *Fusarium* (Zhensheng, 2010), which can infect a variety of crops (Jie et al., 2009). Plant diseases caused by *Fusarium* are serious threats to the sustainable development of agriculture (Gyenis et al., 2003). Studies have shown that 94.5% of wheat scabs are caused by *Fusarium graminearum* (Mohammadi and Kazemi, 2002). Maize ear rot, which is the most detrimental to corn production, is common in major corn-producing regions worldwide, with an incidence of up to 50%, and the main causative pathogen is *Fusarium verticillium* (Cavaglieri et al., 2005). In addition to the food industry, fruit production is endangered by *Fusarium*. For example, *Fusarium* wilt in bananas affects global banana production (Wang et al., 2013). Watermelon wilt caused by *Fusarium* has a severe impact on watermelon production worldwide (Ling et al., 2011; Xue et al., 2019). *Fusarium* is also the main pathogen that causes root rot in Chinese medicinal materials, such as *Panax notoginseng* (Dong et al., 2018), *Atractylodes macrocephala* (Yang et al., 2018), *Chuan Xiong* (Li et al., 2015), and *Astragalus* (Zhao et al., 2009), and severely affects the output and quality of these materials. Wang et al. (2020) reported that the pathogen that causes *Fritillaria* root rot is *Fusarium*, which mainly damages the plant roots. However, no studies have reported the damage caused by *Fusarium* to the bulb of *Fritillaria*.

In recent years, although the use of chemical pesticides to prevent plant diseases has played an important role in agricultural production, the long-term use of large quantities of chemical pesticides has also led to a series of problems by not only increasing the resistance of pathogenic bacteria and insect pests but also making it difficult to control plant diseases (Gerhardson, 2002). Therefore, other ways to ensure competitive food production, provide environmental security, protect plants from pathogens, and maintain the long-term ecological balance of agricultural ecosystems are urgently needed. Biological control is a control method that uses beneficial microorganisms or other organisms in an ecosystem to restrict or eliminate harmful microorganisms and is an integral part of modern agricultural production (Ku Y. L. et al., 2018). At present, microorganisms with biological control ability have been found among different species of bacteria, fungi and actinomycetes (Selosse et al., 2004). Studies have shown that *Pseudomonas, Bacillus* (Karimi et al., 2012), *Trichoderma harzianum* (Saravanakumar et al., 2018), *Streptomyces* (Faheem et al., 2015), etc., can effectively inhibit the growth of *Fusarium*. *Bacillus subtilis* is widely used to control agricultural diseases due to its strong adaptability and good antimicrobial activity in soil (Zhao et al., 2014; Chandrasekaran et al., 2017).

The secretion of some organic acids is one of the methods by which biocontrol bacteria inhibit the growth of pathogens (Lacombe et al., 2010). Benzoic acid has bacteriostatic activity against a variety of pathogenic fungi, with relatively high activity against *Phytophthora infestans* and *Rhizoctonia solani* (Park et al., 2001; Jiashun et al., 2011). Chlorogenic acid permeabilizes the germinating spores and hyphae of pathogens such as *Sclerotinia sclerotiorum*, *Fusarium solani*, *Verticillium dahliae*, and *Botrytis cinerea* (Martínez et al., 2017). Under simulated plant conditions *in vitro*, salicylic acid can inhibit the growth of the grape pathogen *Eutypa lata* (Amborabé et al., 2002).

In this study, we confirmed that the pathogen that caused bulb rot in *F. taipaiensis* P.Y.Li (Taibai Beimu) was *Fusarium.* We explored the ability of *B. subtilis* C3 to inhibit *Fusarium* growth and analyzed the inhibitory mechanism. These findings will help us develop a new biofertilizer to control bulb rot caused by *Fusarium* and contribute to the development of environmentally friendly agriculture.

## Materials and Methods

### Biocontrol Strain and Culture Conditions

*B. subtilis* C3 (GenBank accession number KY983582; the phylogenetic tree of *B. subtilis* C3 is shown in Supplementary Figure 1) was isolated and preserved in our laboratory, and previous studies found that this strain can improve the soil and fruit quality of kiwi in old orchards (Ku Y. L. et al., 2018). The strains were stored in LB liquid medium containing 20% glycerol at −20°C for later use.

### Isolation and Identification of Pathogenic Fungi

Rotten *F. taipaiensis* P.Y.Li bulbs (Supplementary Figure 2) were collected from the experimental base of Taibai Shengfeng Co., Ltd. (107.40°E, 34.06°N), in May 2018. We rinsed the soil from the surfaces of the rotten bulbs with tap water, disinfected the surfaces with 75% alcohol, and washed them 2–3 times with sterile water. Then, we cut sections 0.2–0.3 cm in length from the decayed part and soaked up the moisture on the surface with sterile filter paper. The decayed sections were placed on solid PDA medium and cultured at 30°C. After colonies appeared, we separately picked them onto fresh solid PDA medium for purification. The purified fungus was inoculated into healthy *Fritillaria* to observe its pathogenicity (Yihan et al., 2018). Through the observation of fungal hyphae and spores, the morphological characteristics of each stage were obtained. In addition, fungal genomic DNA was extracted using a fungal genomic DNA extraction kit (spin column type, manufactured by Bioteke Corporation). The sequence of the rDNA ITS region (containing ITS1, 5.8SrDNA, and ITS2) gene was amplified by polymerase chain reaction (PCR) using universal primers (ITS1: 5′-TCCGTAGGTGAACCTGCGG-3′ and ITS4: 5′-TCCTCCGCTTATTGATATGC-3′). The amplified rDNA ITS sequence was aligned and submitted to the National Center for Biotechnology Information (NCBI) GenBank. The target sequences were downloaded from the NCBI official website, and imported into MEGA6.0, together with the sequencing sequences. All sequences were aligned and then saved after their two ends were truncated. Then, the phylogenetic tree was constructed using the neighbor-joining (NJ) method, and the support rate of each node of the phylogenetic tree was repeatedly tested 1000 times with bootstrapping (Sato and Miyazaki, 2017).

### Inhibitory Activity of C3 Against Pathogenic Fungi

*Fusarium* cultured for 4–6 days was inoculated into 20 ml of PDB liquid culture medium, and single C3 colonies of approximately 0.4 cm were added to the medium (the control group was not inoculated with single C3 colonies). The culture was shaken at 180 r/min for 24 h and then transferred to an incubator at 30°C for 4–5 days. The supernatant was filtered with Whatman No. 4 filter paper to obtain hyphae. The hyphae were washed with distilled water and dried in a Petri dish at 80°C for 24 h. Then, we determined the dry weight of the mycelia (m = m_1_−m_2_−m_3_, m is the weight of the mycelia, m_1_ is the weight of petri dish and filter paper after drying, m_2_ is the weight of the Petri dish, and m_3_ is the weight of the filter paper) and calculated the inhibition rate. Each treatment was repeated three times. We picked hyphae for observation under a microscope (Olymous BX53).

### Effect of C3 Fermentation Broth on the Cell Structure of Pathogenic Fungi

After C3 fermentation broth treatment, the *Fusarium* mycelial cells were resuspended in 10 mM PBS (pH 7.2–7.4) and stained with 50 μg/mL propidium iodide (PI) for 20 min at 28°C in the dark. Thereafter, microscopic observation was performed under a fluorescence microscope (Olympus BX53, Japan). Each assay was performed in triplicate, and *Fusarium* mycelia not treated with C3 were used as a control (Zhanga and Suna, 2018).

The DNA condensate of *Fusarium* C3-treated *Fusarium* fungal hyphae was resuspended in 10 mM PBS (pH 7.2–7.4) and stained with 10 μg/mL Hoechst 33258 at 28°C for 20 min in the dark. The staining of *Fusarium* mycelial cells was observed under a fluorescence microscope with a filter (346 nm/460 nm), and *Fusarium* mycelia without C3 were used as a control (Zhanga and Suna, 2018).

### Extraction and Antimicrobial Activity Testing of Crude Antimicrobial Proteins

The methods used for ammonium sulfate sedimentation of the bacteriostatic protein and antimicrobial activity testing were the same as those described by Wang et al. (2016). The C3 seed solution stored at 4°C was inoculated into LB liquid medium at an inoculum of 1%, and shaken at 30°C for 24 h at a rotation speed of 180 r/min to obtain the fermentation broth. The fermentation broth was centrifuged at 4°C, and 10,000 r/min for 10 min, and the precipitate was discarded. Then, the supernatant was precipitated overnight (4°C) with ammonium sulfate at different saturations (50, 60, 70, 80, and 90%), and centrifuged at 4°C and 10,000 r/min for 10 min, and the supernatant was discarded. The precipitate was dissolved in phosphoric acid buffer and dialyzed at 4°C for 48 h. The final dialysis bag contained crude protein extract. The antimicrobial protein was filtered through a 0.22-μm microporous membrane to remove bacteria, and then 100 μL of the filtrate was aspirated and spread evenly onto solid PDA medium. We inserted a mycelial block (ϕ = 6 mm) of the pathogenic fungus *Fritillaria* into the culture medium, setting sterile water as a control, and repeated each treatment 3 times. After incubation at 30°C for 2 days, the colony diameter of the pathogenic fungi was measured by the cross method, and the antimicrobial rate was calculated to determine the optimal ammonium sulfate saturation for extracting the C3 antimicrobial protein. We used the antimicrobial protein with the best ammonium sulfate saturation for SDS-PAGE analysis to determine its molecular weight. 20 μL crude protein solution was added to the injection well and separated by 12% SDS-PAGE. The gel was stained with Coomassie brilliant blue R-250 for 2 h and finally decolorized until clear protein bands were observed.

Chitinase is an enzyme that hydrolyzes fungal cell walls (Cruz et al., 1995). The qualitative determination method of the ability of strain C3 to produce chitinase was as follows. Strain C3 was spotted onto a chitin solid medium plate 4 times, with 3 plates as 3 repetitions. The chitin solid medium formulation was NH_4_H_2_PO_4_ 1.0 g, KCl 0.2 g, MgSO_4_⋅7H_2_O 0.2 g, chitin 10 g, agar 20 g, and deionized water 1000 ml, at pH 7.0. The ability to degrade chitin was determined by whether clear areas appeared around the colonies (Park et al., 2005).

### Active Ingredient Analysis of C3 Fermentation Broth

C3 was inoculated into an inorganic salt solid medium containing bromocresol purple and cultured at 30°C for 24 h. The acid production ability of C3 was determined qualitatively. The C3 seed solution stored at 4°C was inoculated at 1% in acid-producing fermentation medium (10 g/L glucose, 1.5 g/L yeast extract, 0.5 g/L peptone, 0.05 g/L MgSO_4_, 6 g/L CaCO_3_). Fermentation broth was obtained by shaking at 180 r/min and 30°C for 24 h, and C3 fermentation broth was obtained. Blank medium was used as a control, and each treatment was repeated 3 times. The fermentation broth was incubated at 4°C and 10000 r/min for 10 min, and the supernatant obtained was stored until use.

We added concentrated sulfuric acid to 2 mL of the supernatant to adjust the pH to 2–3 to obtain free organic acid in the test solution. 2 mL 2.0 g/L n-propanol was added and mixed as an internal standard. NaCl was added to saturation. Ethyl acetate was added according to the volumetric ratio of the aqueous phase and organic phase (2:1). The mixture was vortexed for 15 s and centrifuged (6000 r/min, 2 min), and the upper organic phase was passed through a 0.22-μm filter membrane for gas chromatography-mass spectrometry (GC-MS, Model number: Shimadzu GC-14C) detection (Fan et al., 2018). The relative acid content was calculated using the peak area normalization method (Schauer et al., 2005). The GC-MS conditions were as follows: the chromatographic column used was a TG-5MS weakly polar column; the injection volume was 1.0 μL; split mode was used; the split flow rate was 33.3 mL/min; and the carrier gas flow rate was 1.0 mL/min. The program temperature was set as follows: the initial temperature was 30°C for 30 min, followed by 2.0°C/min to 100°C for 1 min, 15.0°C/min to 220°C for 1 min, and 30.0°C/min to 300°C for 5 min. An EI detector was used for this measurement, and the detector temperature was 350°C. Compounds were identified by comparing their mass spectra with those of the NIST library^1^ and significant metabolites were identified by comparing their mass spectra and retention indices with those of commercial standards (Adams, 2007). The supernatant was further sterilized by filtering through a 0.22-μm filter membrane and placed in a sample bottle for HPLC analysis. At the same time, we prepared acetic acid, lactic acid, oxalic acid, and citric acid solutions as references. The HPLC (Model number: Waters Alliance HPLC, detector type: fluorescence detector) conditions were as follows: the column used was a Symmetry^®^ C18 (4.6 × 250 mm) column; the injection volume was 10 μL; the column temperature was 25°C; and the mobile phase was methanol (KH_2_PO_4_ (0.02 mol/L) = 3:97, pH 2.5, flow rate 0.6 mL/min).

### Antagonistic Study of the Main Active Ingredients Against Pathogenic Fungi Secreted by C3

Solutions of the main active ingredients were prepared at different concentrations and sterilized by a 0.22-μm filter membrane for use. When the sterilized PDA solid medium had cooled to approximately 40°C, the above solutions of the main active ingredients were added. The liquid medium was poured into a Petri dish and allowed to solidify. Then, the *Fusarium* cake (φ = 6 mm) was placed in the center of the Petri dish. Each treatment was repeated 3 times, and medium without the main active ingredients was used as a blank control. The Petri dish was incubated at 30°C for 5 days. The colony diameter was measured by the cross method to calculate the antimicrobial rate.

### Control Efficacy of C3 for Bulb Rot Disease in *F. Taipaiensis* in a Field Trial

In September 2018, a field experiment was carried out in Tangkou village, Tsuitou town, Taibai County, Shaanxi Province (107.40°E, 34.06°N), and the following treatments were applied: (1) compound fertilizer and (2) compound fertilizer + C3. The test field was divided into 6 plots, each of which was 80 × 130 cm. Two-year-old *F. Taipaiensis* were transplanted at a distance of 8.7 cm × 20 cm and planted by ditching. This experiment used a random monolithic design with three replicates. Soil samples were collected in May, June, and July of the following year, and their soil microbes were determined. The soil microorganisms were diluted by the flat plate method. When harvesting *F. Taipaiensis*, the incidence of bulb rot disease was counted.

### Statistical Analyses

Data entry and analysis were performed using Microsoft Excel 2013. All the data were statistically analyzed using SPSS version 22 (SPSS Inc., Chicago, IL, United States) by one-way analysis of variance (ANOVA). The obtained means were compared by Duncan’s *post hoc* multiple range test and were considered significant at *p* < 0.05. All data acquired from the repeated experiments were expressed as the mean ± standard deviation.

## Results

### Morphological Characteristics and Molecular Identification of Pathogenic Fungi

**FIGURE 1:**
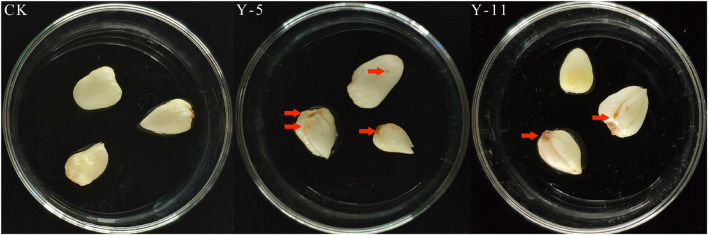
Pathogenic back-testing of *Fritillaria* bulbous pathogens (CK: Sterile water; Y-5: the spore fluid of Fungus named Y-5; Y-11: the spore fluid of Fungus named Y-11. The place pointed by the red arrows is the disease-causing part of the fungus).

Twelve fungal isolates were isolated from rotten *F. taipaiensis* P.Y.Li bulbs and stored and used for the pathogenicity test. Y-5 and Y-11 could be identified as pathogenic fungi by the pathogenicity reconnection test (Figure 1). Seven days later, *Fritillary* bulbs inoculated with Y-5 fungal spore liquid showed disease spots, and the bulb disks turned black. *Fritillary* bulbs inoculated with Y-11 fungal spore liquid also showed the same result, but the pathogenic effect was not as severe as that in the Y-5 inoculated group. The above symptoms were consistent with the symptoms of diseased fritillary bulbs in the field, while the other 10 fungal isolates and controls did not show symptoms similar to those of diseased bulbs. Therefore, the Y-5 and Y-11 fungal isolates were identified as pathogenic fungi.

**FIGURE 2:**
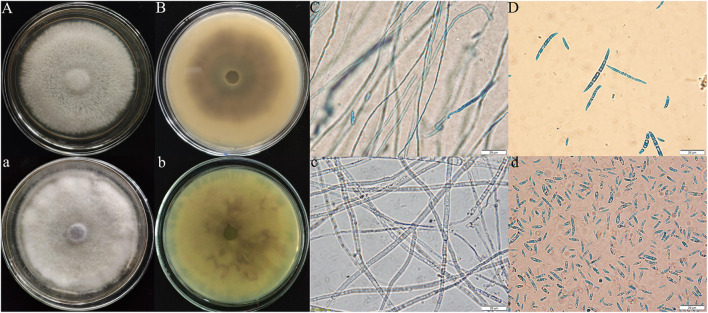
Morphological characteristics of Fritillaria pathogens (Y-5: **A–D**; Y-11: **a–d**; **A,B,a,b**: colony morphology; **C,D,c,d**: microscopic feature).

According to the observation of the colony morphology of the pathogenic fungi, the Y-5 colonies were round, the aerial hyphae were velvety and white with a netted surface, and the backs of the colonies showed purple-red pigmentation, which grew to 9 cm in 7 days. Large conidia of 3–7 compartments were observed under the microscope (Figures 2A–D). The colonies of Y-11 were round, the aerial hyphae were flocculent and white, and the backs of the colonies produced faint purple-red pigmentation in approximately 5 days, which grew to 9 cm in approximately 6 days. A large number of conidia with 2–4 intervals were observed under the microscope (Figures 2a–d).

**FIGURE 3:**
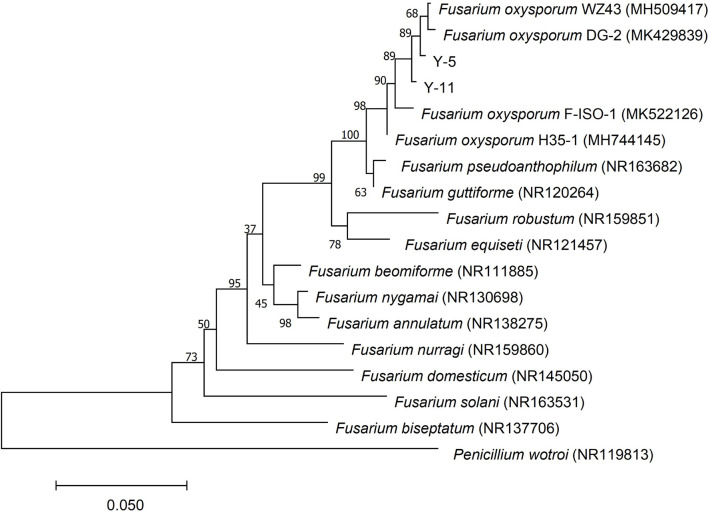
Phylogenetic tree constructed based on ITS sequence of pathogenic fungi Y-5 and Y-11.

The ITS sequence of the pathogenic fungi was compared with the sequences of related strains in GenBank, and the phylogenetic tree shown in Figure 3 was constructed. Y-5, Y-11, and *Fusarium oxysporum* were clustered on a branch with a node support rate of 89%. Comparison with *F. oxysporum* DG-2 (MK429839) and *F. oxysporum* WZ43 (MH509417) showed homologies of 99.44 and 99.25%, respectively; therefore, Y-5 and Y-11 were both judged to be *F. oxysporum*. However, due to the difference in the colony morphology and phylogeny of the two pathogens, we speculated that Y-5 and Y-11 may be two strains of *F. oxysporum*.

### Inhibitory Effect of C3 on Pathogenic Fungi

**FIGURE 4:**
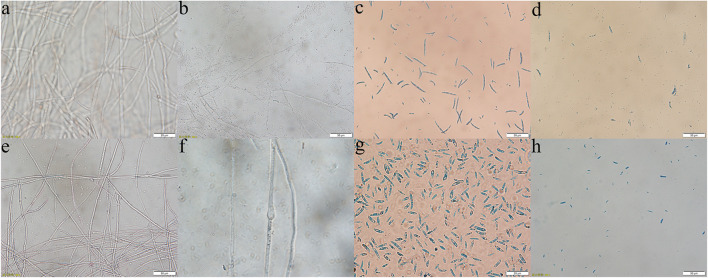
Effect of C3 on pathogenic mycelium and spore morphology (**a–d**: Y-5; **e–h**: Y-11; **a,e,c,g**: mycelium and spores of the control group; **b,f,d,h**: mycelium and spores treated with C3).

Through the liquid cocultivation test, it was found that C3 fermentation broth had a significant inhibitory effect on Y-5 and Y-11 (Supplementary Figure 3). After inoculation with single C3 colonies, the pathogenic mycelia hardly grew normally, and the color of the fermentation broth after cocultivation was clearer than that in the group that was not inoculated with C3. Measurement of the mycelial biomass showed that the inhibition rates of C3 against both Y-5 and Y-11 reached 60%, with values of 64.69 and 69.79%, respectively (Table 1). These results showed that C3 could inhibit the two pathogenic fungi to different degrees. Microscopic observation revealed that the fungal hyphal structure of the control group was intact and grew normally (Figures 4a,e). The amount of sporulation in this group was normal (Figures 4c,g). The fungal hyphae treated with C3 were broken and swollen (Figures 4b,f), and spore production was significantly reduced (Figures 4d,h).

**TABLE 1:** Inhibition effect of C3 on *Fusarium* by liquid co-culture.

Treatments		Dry hypha (mg)	Inhibition rate (%)
Y-5	Y-5	152 ± 0.005	64.69
	Y-5 + C3	54 ± 0.018	
Y-11	Y-11	146 ± 0.009	69.79
	Y-11 + C3	44 ± 0.012	

**FIGURE 5:**
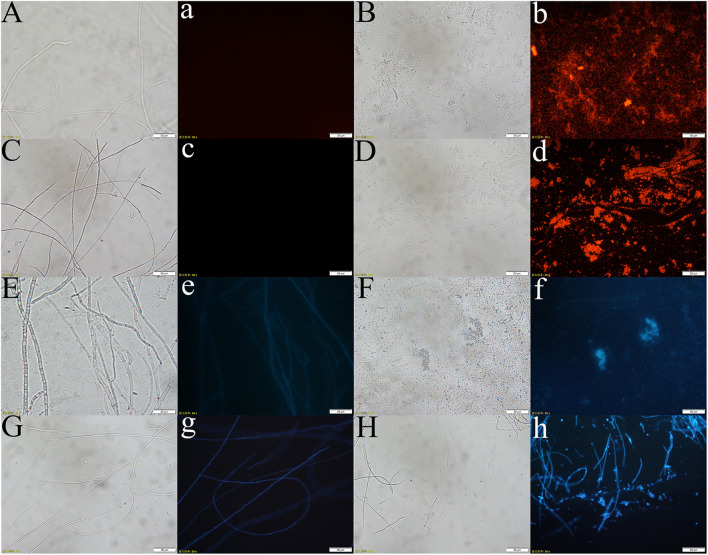
Effect of C3 on the cell integrity and DNA Concentration of Pathogenic fungi (**A–d**: pathogenic fungal hyphae stained with PI; **E–h**: pathogenic fungal hyphae stained with Hoechst 33258. **A–b**, **E–f**: hyphae of funguY-5; **C–d**, **G–h**: hyphae of funguY-11. The two columns on the left are the fungal hyphae of the control group, and the two columns on the right are the fungal hyphae co-cultured with C3).

PI is a fluorescent dye that intercalates into and binds to the base pairs of double-stranded DNA and RNA without base specificity (Rosenberg et al., 2019). PI produces fluorescence upon binding to double-stranded DNA, and the fluorescence intensity is proportional to the content of double-stranded DNA. The hyphal cell viability and cell membrane integrity of pathogenic fungi were examined by PI staining. Y-5 and Y-11 from different treatments were stained with 50 μg/mL PI and observed under a fluorescence microscope. The results showed that C3 induced cell membrane defects and cell death in the Y-5 and Y-11 fungal hyphae. As shown in Figure 5, the cell structures of Y-5 and Y-11 that were not cocultured with C3 were basically complete (Figures 5A,C) and showed weak red fluorescence (Figures 5a,c). However, Y-5 and Y-11 cocultured with C3 did not have a complete cell structure (Figures 5B,D). The cell membrane was destroyed, the protoplast was exuded, and the exuded cell nucleus showed strong red fluorescence (Figures 5b,d).

During cell death, chromatin undergoes a phase change from a heterogeneous, genetically active network to an inert, highly condensed form (Ciccia and Elledge, 2010). Nuclear chromatin condensation, along with concomitant DNA fragmentation, is one of the most important criteria used to identify apoptotic cells. When stained with DNA-binding nuclear dyes, compacted chromatin appears brighter than chromatin in non-apoptotic cells, and condensed nuclei can be easily identified by fluorescence microscopy. Hoechst 33258 is a blue fluorescent dye that penetrates cell membranes and is often used for apoptosis detection. Hoechst 33258 (10 μg/mL) was used to stain Y-5 and Y-11 from different treatments to explore the effects of C3 on their chromatin. As shown in Figure 5, the hyphae that were not treated with C3 exhibited faint blue fluorescence (Figures 5e,g). In contrast, the C3-treated hyphae of Y-5 displayed strong blue fluorescence, which was indicative of chromatin condensation and DNA fragmentation (Figure 5f). Although the C3-treated hyphae of Y-11 showed a hyphal structure (Figure 5H), we also observed strong blue fluorescence, indicating that the cells were dead and the chromatin was condensed (Figure 5h).

### Antimicrobial Activity of Crude Antimicrobial Proteins Secreted by C3

The inhibitory effects of crude protein precipitated by ammonium sulfate at different saturations on pathogenic fungi were quite different. As shown in Table 2, when the ammonium sulfate saturation was 50%, the precipitated protein had the weakest inhibitory effect on the two pathogenic fungi Y-5 and Y-11, with inhibition rates of 25.53 and 24.34%, respectively. As the saturation of ammonium sulfate increased, the inhibition rate increased. When the saturation of ammonium sulfate was 80%, the inhibition rate peaked, with values of 52.48 and 50.00% for Y-5 and Y-11, respectively. However, as the saturation of ammonium sulfate continued to increase, the inhibition of pathogenic fungi weakened. Therefore, the optimal ammonium sulfate saturation for inhibiting pathogenic fungi was 80%. The C3 fermentation broth and the antimicrobial protein extract obtained by 80% ammonium sulfate precipitation were analyzed by SDS-PAGE. Both contained proteins with a relative molecular mass of approximately 50 kD, and the bands of the antimicrobial proteins extracted by 80% ammonium sulfate precipitation were very clear (Supplementary Figure 4). Therefore, the protein with a molecular mass of approximately 50 kD was the main antimicrobial protein of the biocontrol bacterium C3.

**TABLE 2:** Inhibition of Pathogenic fungi by different saturation antibacterial proteins.

Counts of C3 (cfu/g fw)	Ammonium sulfate saturation	Y-5		Y-11	
		Colony diameter (cm)	Inhibition rate (%)	Colony diameter (cm)	Inhibition rate (%)
5.5 ^∗^10^9^	CK	3.53 ± 0.25a	−	3.80 ± 0.14a	−
	50%	2.63 ± 0.04b	25.53	2.88 ± 0.11b	24.34
	60%	2.30 ± 0.00c	34.75	2.23 ± 0.11c	41.45
	70%	2.30 ± 0.07c	34.75	2.10 ± 0.07cd	44.74
	80%	1.68 ± 0.11e	52.48	1.90 ± 0.14d	50.00
	90%	1.98 ± 0.04d	43.97	1.95 ± 0.07d	48.68

*Values are mean ± SE of three replicates.*

*Within each vertical column, values followed by the same letter are not statistically different, according to Fisher’s protected LSD (P < 0.05).*

### Determination of Active Ingredients in the C3 Fermentation Supernatant

**FIGURE 6:**
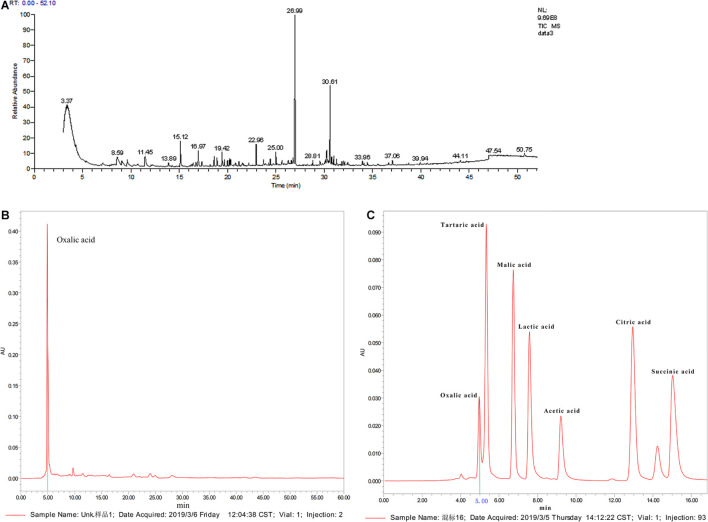
Active ingredient analysis of C3 fermentation broth by GC-MS and HPLC (**A:** qualitative analysis on the ethyl acetate extracted samples by GC-MS; **B:** organic acid standard by HPLC analysis; **C:** C3 fermentation supernatant by HPLC analysis).

We carried out qualitative analysis of the ethyl acetate-extracted samples by GC-MS combined with computational retrieval to separate and identify the chemical components (Figure 6A). A total 105 substances were detected in the supernatant extract. The main types of chemical components were organic acids, esters, benzene and its derivatives, alkanes, ketones, heterocyclic compounds, etc. We compared the eight compounds with the highest contents with the mass spectrometry and retention indices of commercial standard products and found that they were mainly esters and ketones (Supplementary Table 1). Therefore, ethyl palmitate and paeonol, which had the highest contents in the two species, were selected for the fungal inhibition test.

Supplementary Figure 5 shows that C3 could secrete organic acids. pH measurements showed that the pH of the C3 fermentation broth was 6.83, which was 0.42 lower than that of the blank medium. Since water-soluble short-chain organic acids were not detected in the C3 fermentation broth by GC-MS, further qualitative analysis by HPLC was conducted (Figure 6A). The standard chromatogram of organic acids is shown in Figure 6B, and the chromatogram of the C3-secreted organic acids is shown in Figure 6C. It is obvious from the above 2 figures that only oxalic acid and acetic acid were detected among the 7 water-soluble small-molecule organic acids, and the content of oxalic acid was relatively high. Therefore, oxalic acid was considered one of the key organic acids secreted by C3.

### Inhibition of Pathogenic Fungi by the Main Active Ingredients in the C3 Fermentation Supernatant

All the main active ingredients inhibited the growth of the pathogenic fungi Y-5 and Y-11 (Supplementary Figures 6, 7). As the concentration of the main active ingredients increased, the inhibitory effect on the fungi increased. As shown in Table 3, when the concentration of paeonol reached 1.0 mg/mL or the concentration of ethyl palmitate reached 2.0 mg/mL, the Y-5 and Y-11 hyphae hardly grew. When the paeonol concentration was higher than 0.05 mg/mL, the treatment group supplemented with paeonol showed a significant difference (*P* < 0.05) from the control group. When the paeonol concentration was 0.5 mg/mL, the inhibition rates of Y-5 and Y-11 were greater than 60%, with values of 61.11 and 66.67%, respectively (Table 3). Compared with the control group, the ethyl palmitate treatment group showed a significant difference (*P* < 0.05). When the concentration of ethyl palmitate was 0.5 mg/mL, the inhibition rates of Y-5 and Y-11 were only 22.86 and 19.48%, respectively. When the concentration was 1.0 mg/mL, the inhibition rates of Y-5 and Y-11 were 37.60 and 34.96%, respectively. In summary, at the same concentration, paeonol had a better inhibitory effect on Y-5 and Y-11 than ethyl palmitate. Compared with the control group, the treatment groups treated with different concentrations of oxalic acid also showed a significant difference (*P* < 0.05). When the final concentration of oxalic acid was 10 mmol/L, the inhibition rates of Y-5 and Y-11 reached 57.61 and 57.14%, respectively. Oxalic acid played an important role in the suppression of the pathogenic fungi Y-5 and Y-11 (Table 3).

**TABLE 3:** Inhibition of pathogenic fungi by different concentrations of main active ingredients.

	Oxalic acid			Paeonol			Ethyl palmitate		
Pathogen	Concentration (mmol/L)	Colony diameter (cm)	Inhibition rate (%)	Concentration (mg/mL)	Colony diameter (cm)	Inhibition rate (%)	Concentration (mg/mL)	Colony diameter (cm)	Inhibition rate (%)
Y-5	0	6.90 ± 0.14a	–	0	7.20 ± 0.14a	–	0	7.20 ± 0.14a	–
	2.5	5.28 ± 0.18b	23.55	0.05	6.73 ± 0.04b	6.6	0.5	5.55 ± 0.26b	22.86
	5	3.85 ± 0.08c	44.20	0.2	5.28 ± 0.04c	26.74	1.0	4.49 ± 0.33c	37.60
	10	2.93 ± 0.11d	57.61	0.5	2.80 ± 0.00d	61.11	1.5	2.90 ± 0.00d	59.7
				1.0	0.8 ± 0.00e	88.10	2.0	1.50 ± 0.00e	79.10
Y-11	0	5.95 ± 0.07a	–	0	7.28 ± 0.04a	–	0	7.28 ± 0.04a	–
	2.5	4.98 ± 0.11b	16.39	0.05	6.55 ± 0.71a	9.97	0.5	5.86 ± 0.21b	19.48
	5	3.55 ± 0.00c	40.34	0.2	5.05 ± 0.21b	30.58	1.0	4.65 ± 0.09c	34.96
	10	2.55 ± 0.07d	57.14	0.5	2.43 ± 0.04c	66.67	1.5	3.12 ± 0.00c	57.32
				1.0	0.8 ± 0.00d	87.79	2.0	0.8 ± 0.00d	77.56

*Values are mean ± SE of three replicates. Within each vertical column, values followed by the same letter are not statistically different, according to Fisher’s protected LSD (P < 0.05). The same as below.*

### Biocontrol Efficacy of C3 on Bulb Rot Disease of *F. taipaiensis* in a Field Trial

In the field experiment, the number of bacteria and fungi in the soil of *F. taipaiensis* was counted (Table 4). The results showed that after the application of strain C3, the number of bacteria in the soil was higher than that of the control in the three periods, and reached a significant level in the latter two periods. The number of soil fungi in the C3 treatment group was lower than that in the control group in May and July. The ratio of bacteria to fungi in the C3 group was higher than that in the CK group in the three periods. The application of C3 changed the soil microbial community structure of *Fritillaria* and reduced the number of fungi in the soil. The incidence rate of C3 bulb rot in *Fritillaria* at harvest was 28.3%, which was 6.4% lower than that in the control group (Table 4). After applying C3, the average fresh weight of *Fritillaria* bulbs increased. Therefore, strain C3 not only had an obvious control effect on bulb rot in *Fritillaria*, but also improved the biomass of *Fritillaria.*

**TABLE 4:** Effect of C3 on Soil Microorganisms of *F. Taipaiensis*.

Time	Treatments	Bacterial (10^6^ cfu/g fw)	Fungus (10^4^cfu/g fw)	Bacterial- fungus ratio (100)	Incidence of bulb rot disease (%)	Average fresh weight (mg)
219.5.15	CK	7.5 ± 2.121a	3.5 ± 0.707a	2.143	−	−
	C3	10.4 ± 0.707a	2.2 ± 0a	4.727	−	−
2019.6.3	CK	23.5 ± 0.707b	11 ± 1.414a	2.136	−	−
	C3	38.7 ± 12.021a	13.8 ± 1.414a	2.804	−	−
2019.7.12	CK	23 ± 2.828^b^	14 ± 1.414a	1.643	34.7	505.45a
	C3	110.6 ± 14.142a	13.46 ± 2.121a	8.21	28.3	541.98a

## Discussion

Related literature reports on bulb rot disease in *Fritillaria* have been published. In 1987, pathogenic organisms causing bulb rot in *Fritillaria* in Hubei, China, were first reported as *Fusarium avenaceum* [*Gibberella avenacea*] and *Erwinia carotovora* (Liao et al., 1987). However, Wu et al. (1989) reported that the bulb mite is the main transmitter of Anhui fritillary rot and seriously damages the fritilla. Riziwanguli et al. (2011) stated that the causative pathogen of Xinjiang Siberian *fritillary* bulb rot is *S. rapulum* Boull. In our study, after collecting rotted bulbs of *F. taipaiensis*, separating and purifuing the pathogenic fungi, detecting the pathogenicity, and morphologically observing and molecularly identifying the fungi, we found that the fungus responsible for bulb rot in *F. taipaiensis* was *F. oxysporum*. This is the first report on the causative pathogen of bulb rot in *F. taipaiensis* in Shaanxi Province.

*Fusarium* is a complex genus of ascomycete fungi that consists of plant pathogens of agricultural relevance. Controlling *Fusarium* infection, which leads to substantial yield losses, in crops is a major challenge. These economic losses, along with environmental and human health-related concerns regarding the usage of chemicals for disease control, are shifting focus toward the use of biocontrol agents for effective control of phytopathogenic *Fusarium* spp. (Khan et al., 2018). Biological control of plant diseases has always been a hot topic in the agricultural field, and good results have been achieved (Howell, 2003; Alabouvette et al., 2006; Feng et al., 2021). The rhizobacterium *B. subtilis* is considered one of the most widely used and well-studied biocontrol organisms, and 4–5% of its genome is responsible for the synthesis of antibiotics such as the cyclic lipopeptides (LPs) surfactin, iturin, and fengycin (Stein, 2005). In a liquid coculture experiment, we found that *B. subtilis* C3 could effectively inhibit the growth of *F. oxysporum*, and the inhibition rate was up to 60%. *B. subtilis*, as an important biocontrol microorganism, has strong competitive viability, and it competes with *Fusarium* for nutrients or secretes antimicrobial substances to inhibit *Fusarium* (Kloepper et al., 2004; Yu et al., 2018). Our research results showed that after cocultivation with the C3 strain, the mycelial cells of the pathogenic fungus swelled, the cell structure was destroyed, the integrity of the cell membrane was damaged, the protoplasts leaked, and the production of conidia by the pathogenic fungus was suppressed. The fungal cell wall can be regarded as the “armor” of the cell, protecting the fungal cell from changes in osmotic pressure and other environmental effects (Latgé, 2007), and the fungal plasma membrane is mainly responsible for maintaining the order and integrity of the cell (De Ac On, 2013). In Supplementary Figure 8, there are clear zones around the colony of strain C3, indicating that strain C3 produced chitinase which destroyed fungal cell walls (Luo et al., 2015; Schnbichler et al., 2020). Fungal cell wall deconstruction is viewed as a key element of fungal antagonism and therefore of antifungal biocontrol in general. Studies have shown that antimicrobial substances can interact with fungal plasma membranes, after which the cell wall integrity is destroyed, and cell protoplast leakage is induced, causing fungal cell death (Deleu et al., 2008; Gong et al., 2015). Chakraborty et al. (2020) demonstrated the inhibition of conidiogenesis by linear LPs from a strain of *B. subtilis*. Similar to these results, strain C3 inhibited the growth of pathogenic fungi by destroying the cell structure and inhibiting conidiogenesis.

Chromatin concentration and DNA fragmentation are important methods for identifying apoptosis (Tang et al., 2014). To further explore how C3 inhibits the growth of pathogenic fungi, we designed a mycelial staining test. The results of the staining test showed that the pathogenic fungi exhibited chromatin condensation and DNA fragmentation, thereby confirming cell death. A previous research showed that cyclic LPs produced by B. subtilis BS155 metabolism caused chromatin condensation in fungal hyphal cells, which led to the upregulation of DNA repair-related protein expression and the cleavage of poly (ADP-ribose) polymerase (Zhanga and Suna, 2018). Antifungal compounds in the culture supernatant produced by *B. subtilis* V26 can destroy the cell wall of *R. solani* and cause pathogenic mycelia to appear highly vacuolated, exhibiting protoplasm leakage, irregular growth, distortion, and breakage (Wu et al., 2019). These results were consistent with the results of this study, in which the C3 fermentation supernatant destroyed the cell structure of the pathogenic fungi, leading to chromatin concentration and eventually cell death.

The antimicrobial proteins of *Bacillus* mainly include bacteriocins, cell-wall-degrading enzymes and some unidentified antimicrobial proteins. Bacteriocin is a low-molecular-weight protein synthesized by bacteria that has an antibiotic effect on other microorganisms. LPs probably represent the most common class of bacteriocins produced by *Bacillus* spp. (Zeriouh et al., 2011) and are divided into three families: surfactins, iturins, and fengycins (Ongena and Jacques, 2008). In addition to direct antimicrobial activity, LPs may also promote plant disease control by inhibiting the biofilms formed by pathogens or by inducing plant systemic resistance (Massomo et al., 2010; Ongena et al., 2010; Guo et al., 2014; Fan et al., 2017). The relative molecular masses of these three bacteriostatic proteins were all below 2 kD. However, this study found that the relative molecular mass of the antagonistic protein that had the best inhibitory effect on the bulb rot pathogen *Fritillaria vulgaris*, was 50 kD, and this protein was a macromolecular protein. Liang et al. (2005) found that the relative molecular masses of the antagonistic proteins produced by *B. subtilis* B110 that inhibited cotton *Fusarium* wilt were 50 and 29 kD. A protein secreted by *Bacillus velezensis* with a molecular weight of approximately 50 kD could inhibit the growth of *V. dahliae* hyphae and the germination of spores (Liu and China, 2011). Therefore, the protein with a molecular mass of 50 kD was a key antimicrobial protein, and further purification of its active components will be very valuable.

There have been many reports showing that *B. subtilis* effectively inhibits the growth of *Fusarium* (Chang et al., 2010; Tan et al., 2013; Xie et al., 2015). *B. subtilis* secondary metabolites that inhibit pathogenic fungi include volatile compounds, organic acids, and other chemical substances (Kubo et al., 2004; Akram et al., 2015; Zhang et al., 2020). By GC-MS and HPLC analyses, we examined whether other components in the fermentation broth could inhibit the growth of pathogenic fungi. The main active ingredients produced by *B. subtilis* C3 were paeonol, ethyl palmitate, and oxalic acid, and the plate antimicrobial test showed that these three active ingredients inhibited the pathogen *F. oxysporum* to varying degrees. Previous studies have shown that paeonol not only has a freshness-maintaining effect on fruits and vegetables but also inhibits plant insect pests (Kang and Shang, 2007; Jiang et al., 2015). Organic acids inhibit the growth of fungi (Kang and Go, 2002), and oxalic acid is one such acid. At a certain concentration, oxalic acid directly inhibits fungal hyphae and spores (Chet, 1998; Nagy et al., 2012). Many research results have confirmed that acid esters (4-hydroxybenzoic acid esters, caffeic acid esters, fatty acid methyl esters, etc.) prevent fungal growth (Ravn et al., 1989; Vol, 1994; Vincent, 2010), and ethyl palmitate is one of them (Chandrasekaran et al., 2011). The three active ingredients among the secondary metabolites of *B. subtilis* had a certain inhibitory effect on *F. oxysporum*, which was consistent with the above research results. Our results, for the first time, demonstrated that the pathogenic fungus that causes *F. taipaiensis* bulb rot is *F. oxysporum*. *B. subtilis* C3 inhibited conidiogenesis of *F. oxysporum* and destroyed the cell structure of its hyphae, causing protoplast exudation, chromatin concentration, DNA fragmentation, and, ultimately, cell death. The antimicrobial protein and main active components among the secondary metabolites of C3 could inhibit the growth of *F. oxysporum*. This work will provide novel guidance for the biological control of *F. taipaiensis* bulb rot. The field test results in the *F. taipaiensis* planting area showed that the incidence of *Fritillaria* bulb rot disease was reduced after the application of strain C3, indicating that the strain C3 can indeed control *F. taipaiensis* bulb rot disease. The results of our preliminary indoor experiments have been effectively verified.

## Data Availability Statement

The original contributions presented in the study are included in the article/Supplementary Material, further inquiries can be directed to the corresponding author.

## Author Contributions

CC and PP designed and supervised this study. YK, NY, PP, XM, and LC performed the experiments. YK and NY analyzed the data and drafted and revised the manuscript. All authors read and approved the manuscript for submission.

## Conflict of Interest

The authors declare that the research was conducted in the absence of any commercial or financial relationships that could be construed as a potential conflict of interest.

## Publisher’s Note

All claims expressed in this article are solely those of the authors and do not necessarily represent those of their affiliated organizations, or those of the publisher, the editors and the reviewers. Any product that may be evaluated in this article, or claim that may be made by its manufacturer, is not guaranteed or endorsed by the publisher.

## Footnotes

1
https://webbook.nist.gov/chemistry/
