# Corrigendum: Biocontrol Mechanism of *Bacillus subtilis* C3 Against Bulb Rot Disease in *Fritillaria taipaiensis* P.Y.Li

**DOI:** 10.3389/fmicb.2022.855980

**Published:** 2022-02-09

**Authors:** Yongli Ku, Nan Yang, Peng Pu, Xueli Mei, Le Cao, Xiangna Yang, Cuiling Cao

**Affiliations:** ^1^College of Life Sciences, Northwest A&F University, Yangling, China; ^2^College of Chemistry and Pharmacy, Northwest A&F University, Yangling, China; ^3^College of Environment and Life Sciences, Weinan Normal University, Weinan, China

**Keywords:** *Fritillaria taipaiensis* P.Y.Li, *Bacillus subtilis*, *Fusarium*, active ingredients, biological control

In the published article, there was an error in affiliations. For author Yongli Ku, instead of “College of Forestry, Northwest A&F University”, it should be “College of Life Sciences, Northwest A&F University”.

The authors apologize for this error and state that this does not change the scientific conclusions of the article in any way. The original article has been updated.

## Publisher's Note

All claims expressed in this article are solely those of the authors and do not necessarily represent those of their affiliated organizations, or those of the publisher, the editors and the reviewers. Any product that may be evaluated in this article, or claim that may be made by its manufacturer, is not guaranteed or endorsed by the publisher.

