# Supplementary material for: Biocontrol Mechanism of Bacillus subtilis C3 Against Bulb Rot Disease in Fritillaria taipaiensis P.Y.Li
Source: Front Microbiol. 2021 Sep 30;12:756329. doi: 10.3389/fmicb.2021.756329 (PMC8515143; doi:10.3389/fmicb.2021.756329)
Supplement: Supplementary file 1 [file Data_Sheet_1.docx]

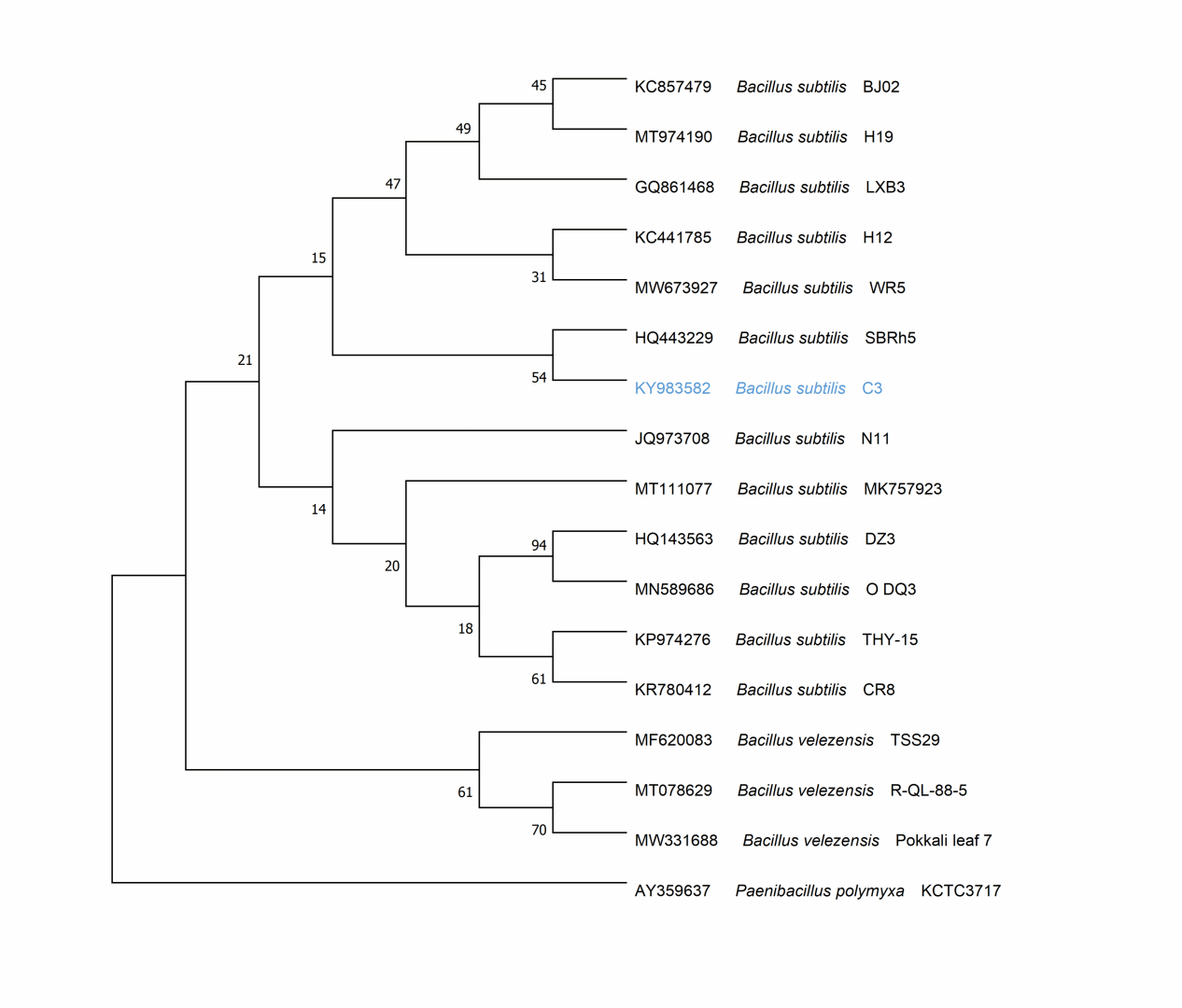


Fig.S1 The phylogenetic tree of *B. subtilis* C3


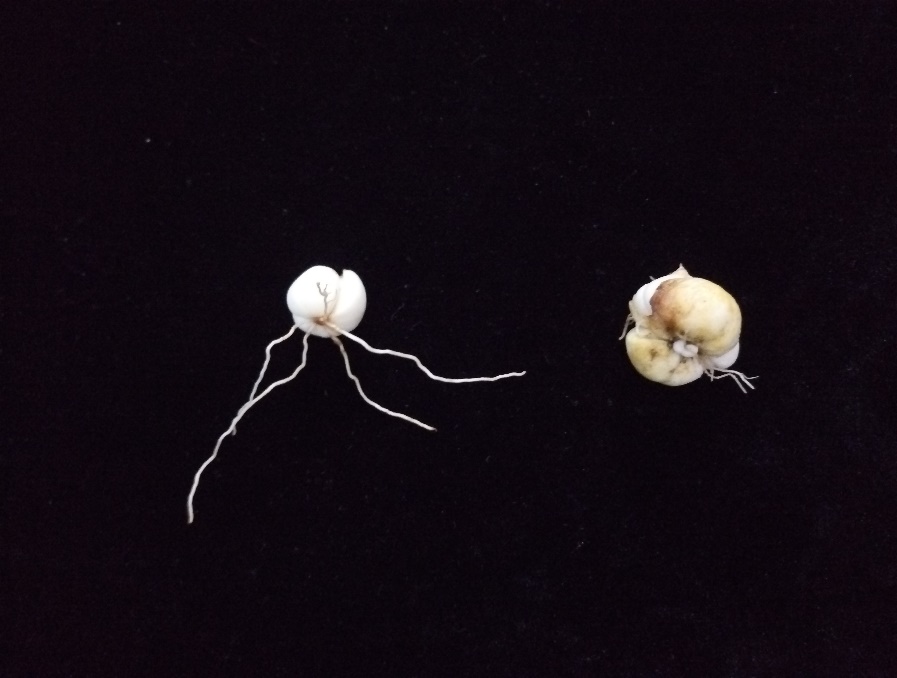


**a**


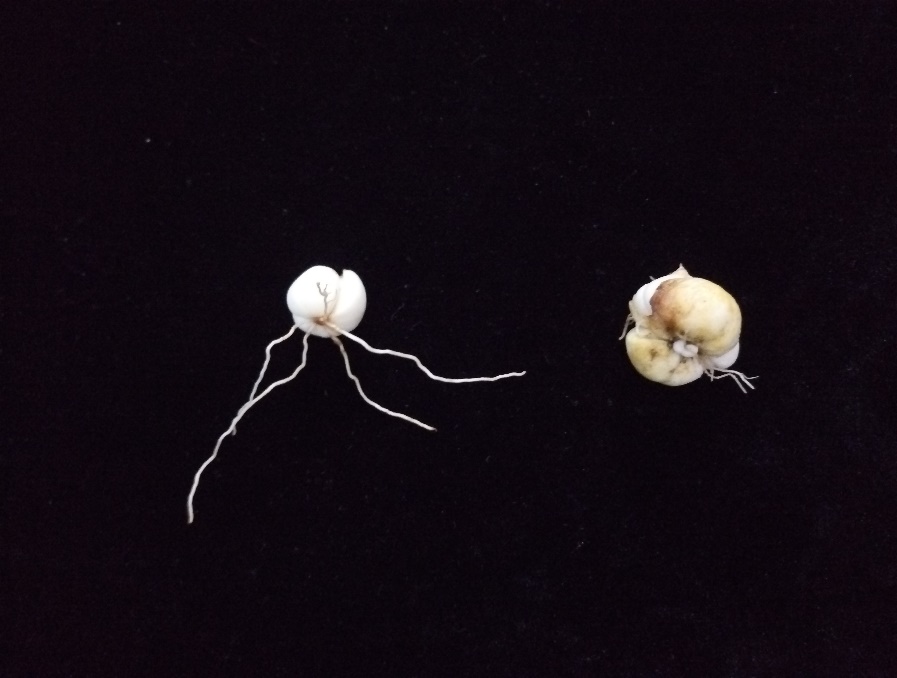


**d**


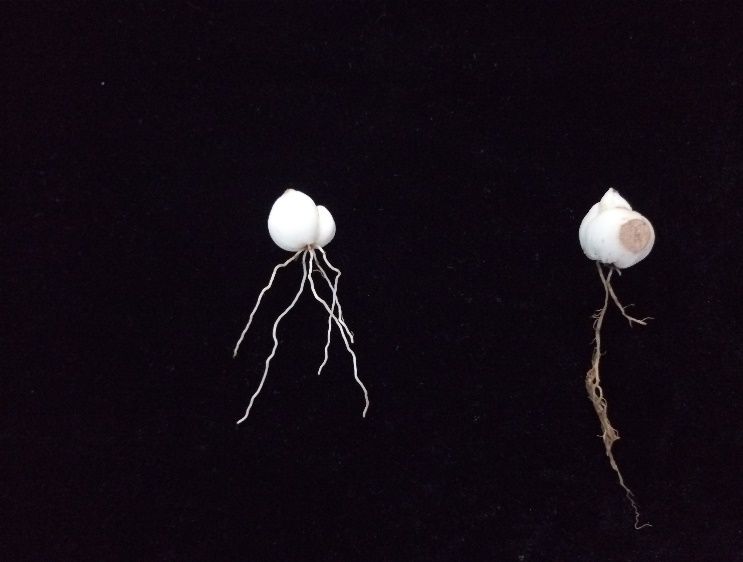


**b**


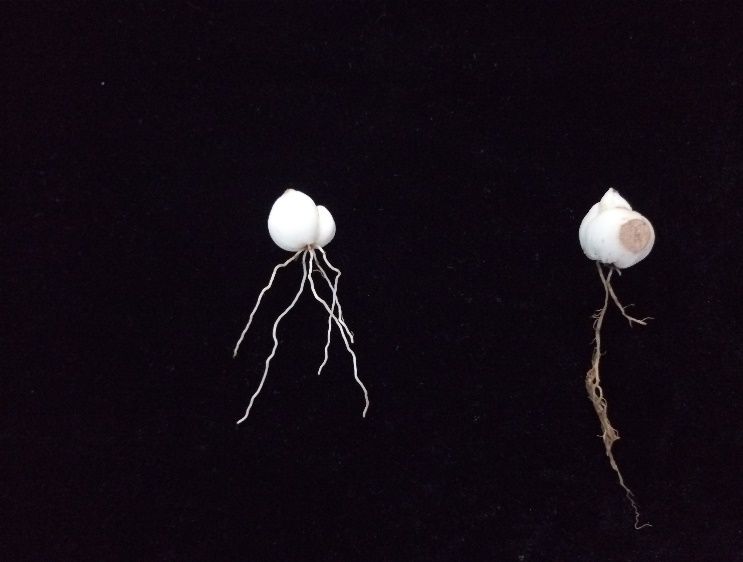


**e**


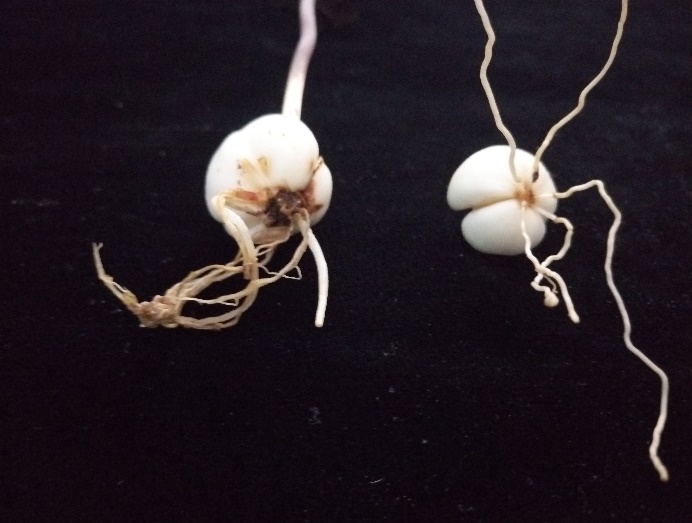


**c**


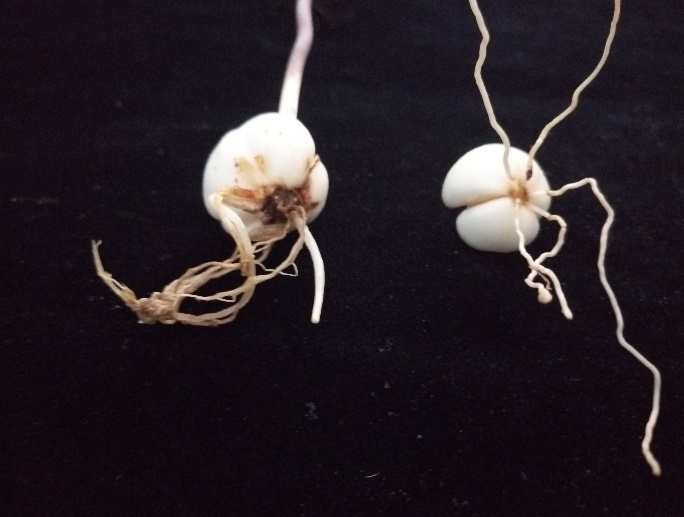


**f**

Fig.S2 Symptoms of perennial *Fritillaria* bulb rot (a, b, c: Healthy bulb;d, e, f: Diseased bulb)


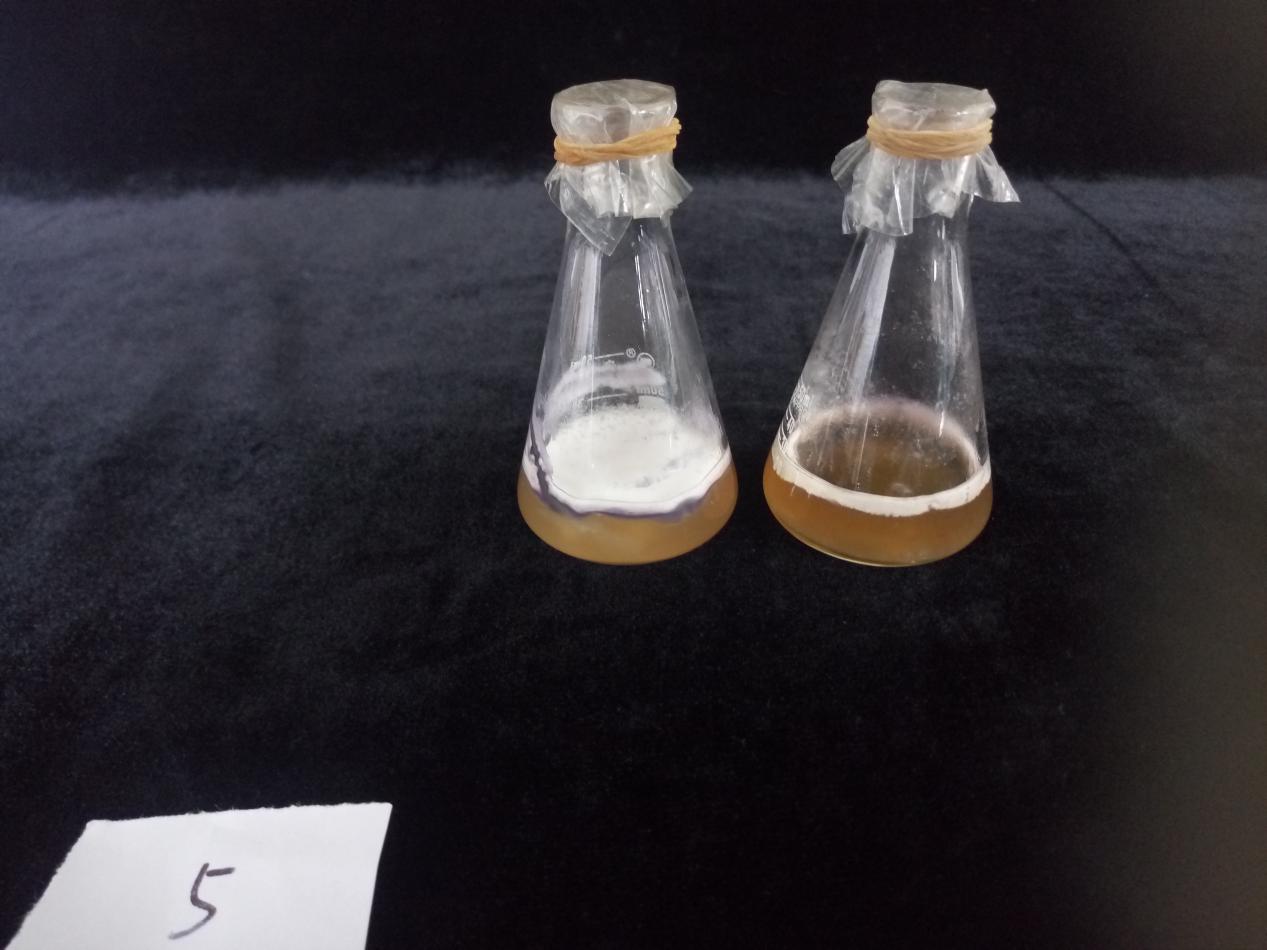

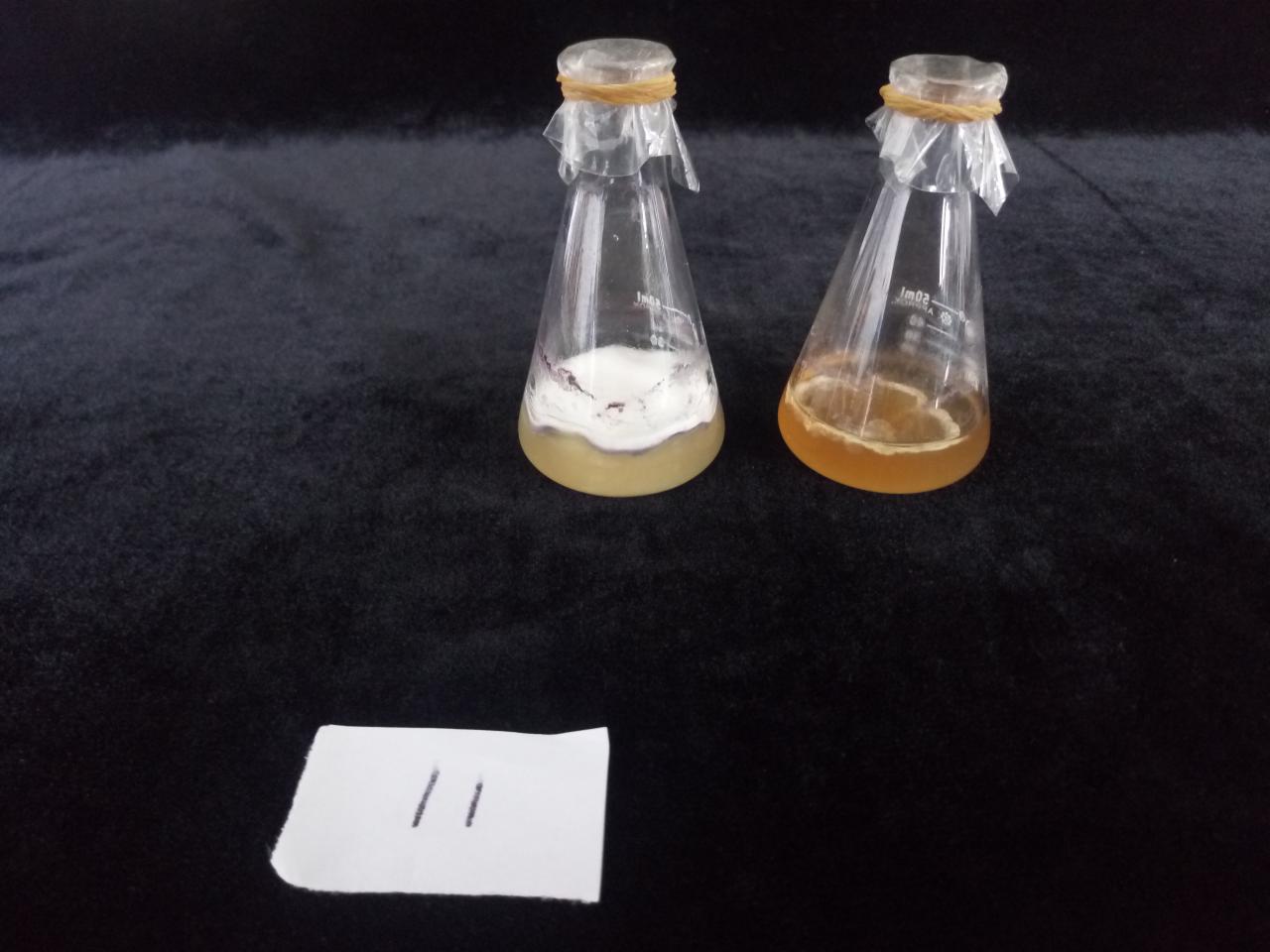


**A**

**B**

Fig.S3 Inhibition effect of C3 on *Fusarium* by liquid co-culture

(A: Y-5 (Left: CK; Right: Treated with C3); B: Y-11 ( Left: CK; Right: Treated with C3))

M


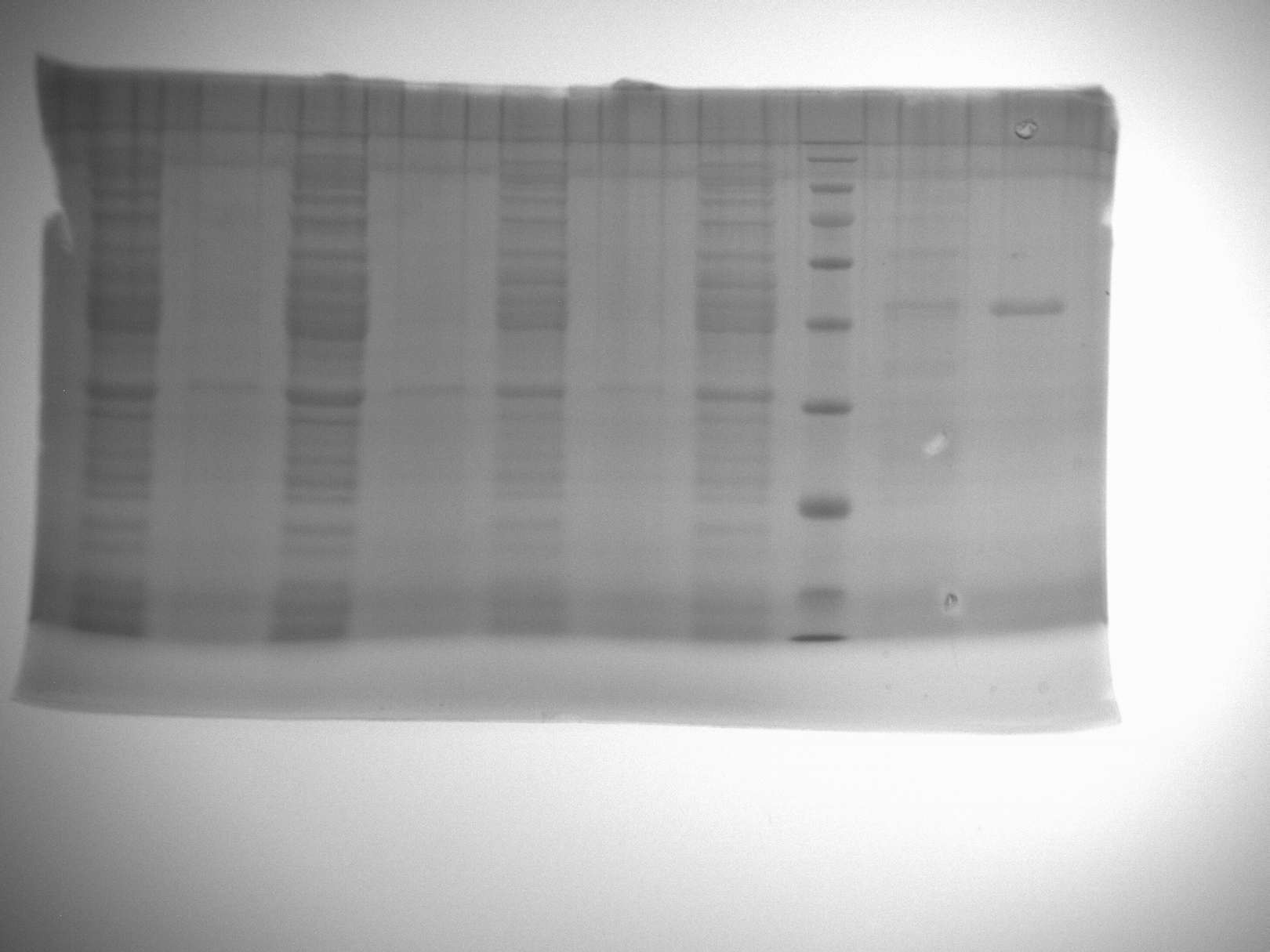


C3

80%P

45 kDa

65 kDa

Fig.S4 Coomassie brilliant blue R-250 stained SDS-PAGE. M: broad range protein markers; C3: filtered C3 fermentation broth; 80%P: crude proteins fractionated with 80% (NH_4_)_2_SO_4._ Numbers on the left side of the gels correspond to the molecular weight of the markers.


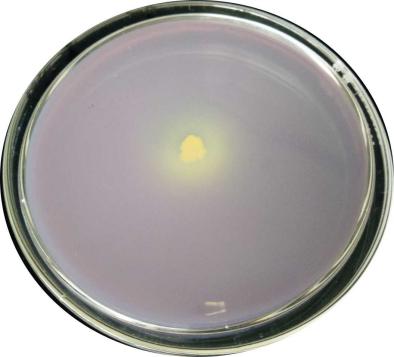


Fig.S5 Organic acids secreted by C3


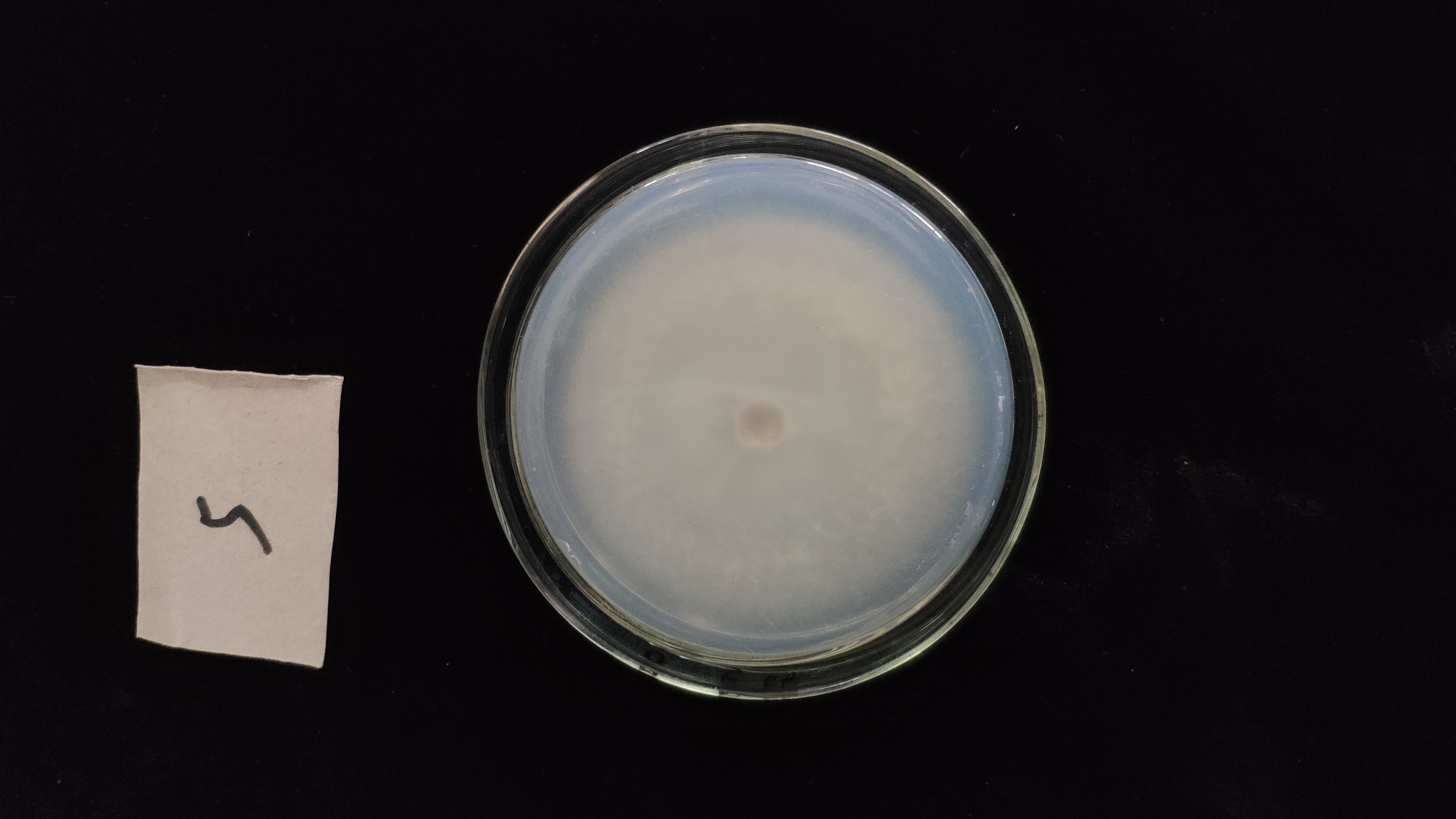

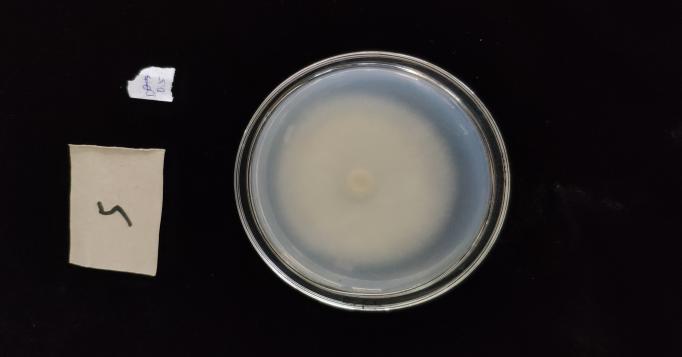

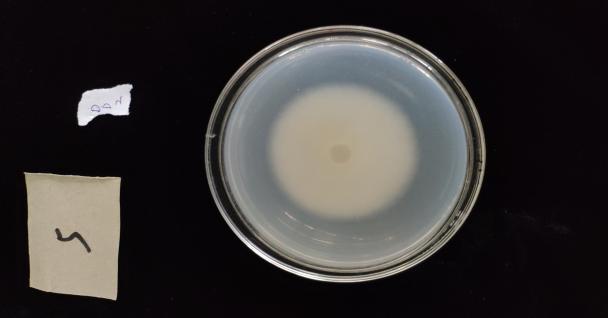

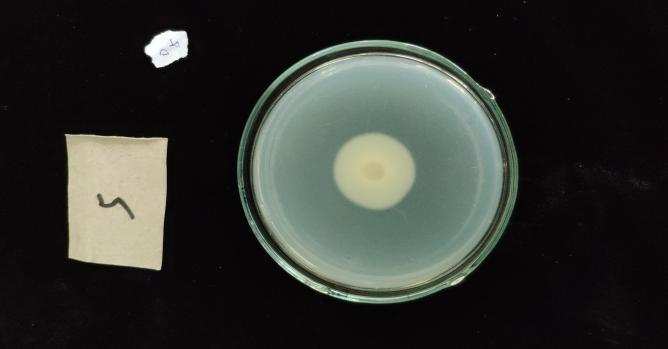

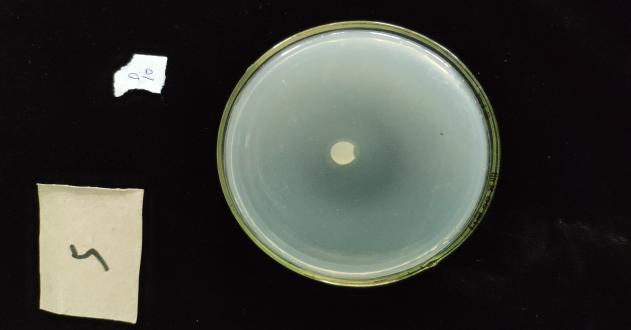

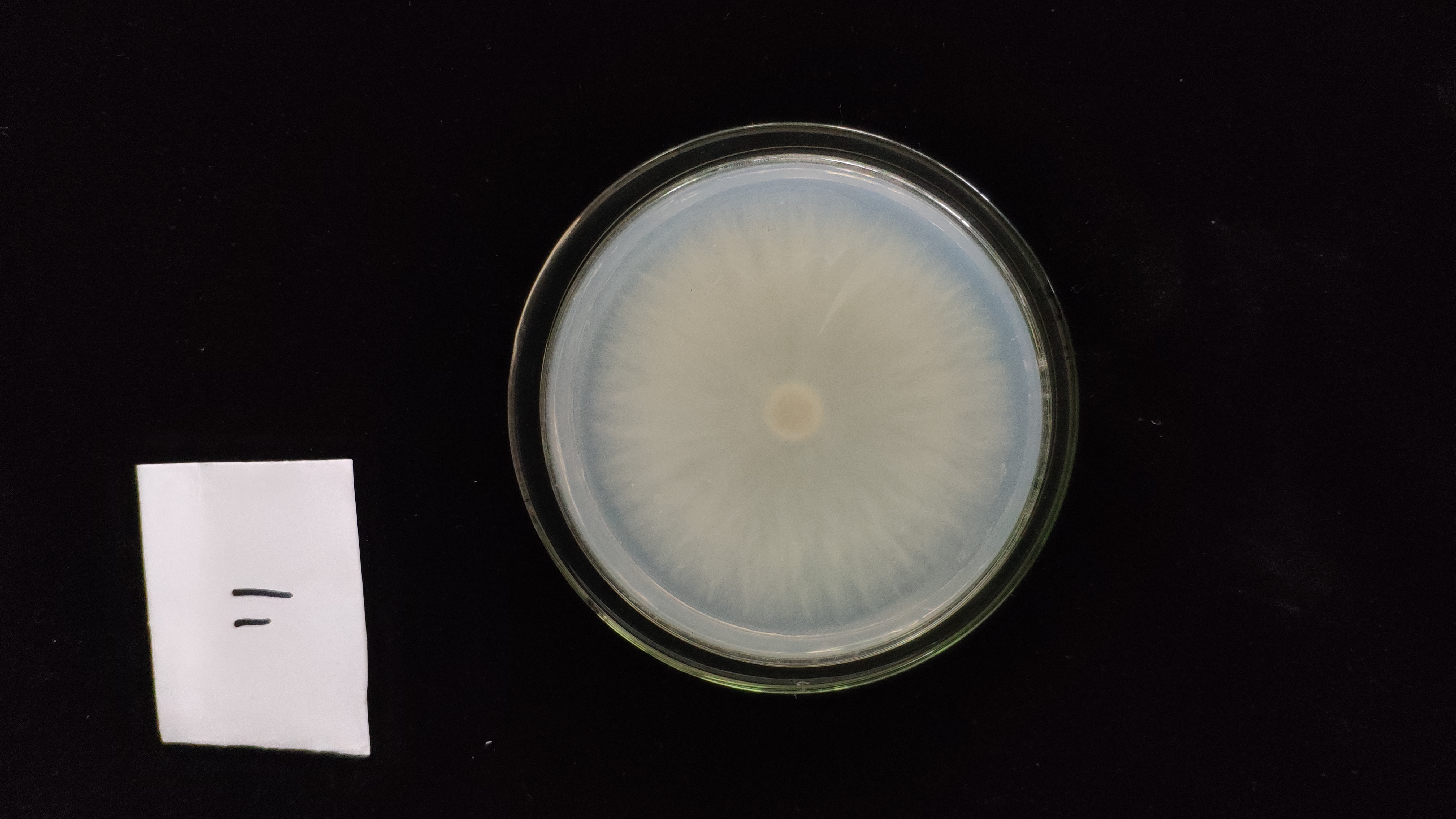

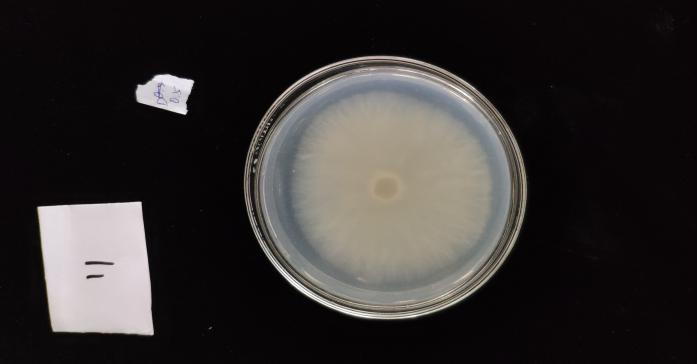

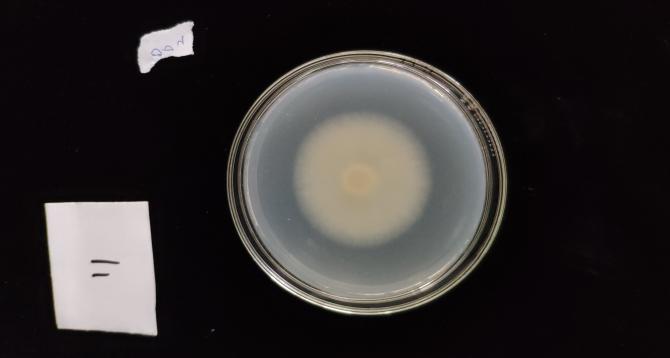

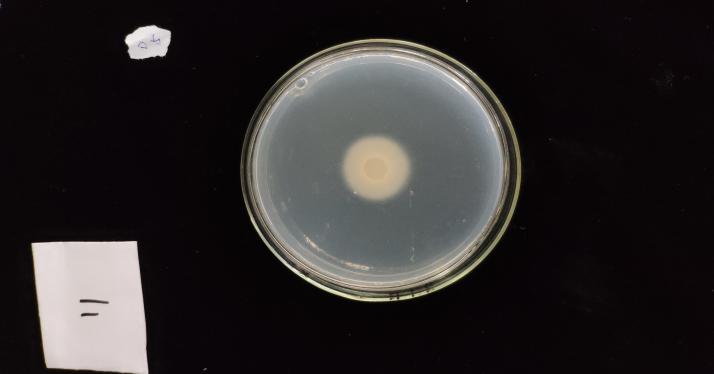

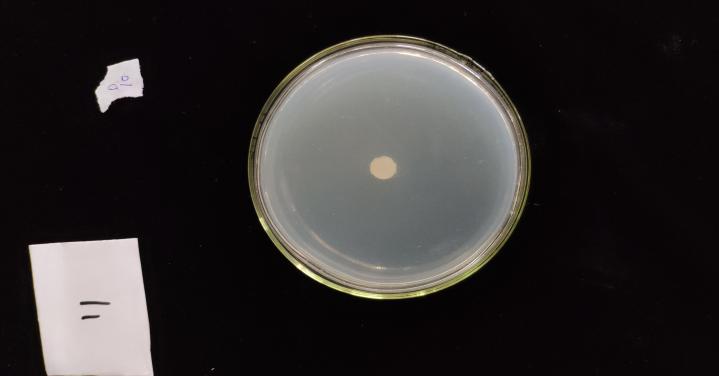

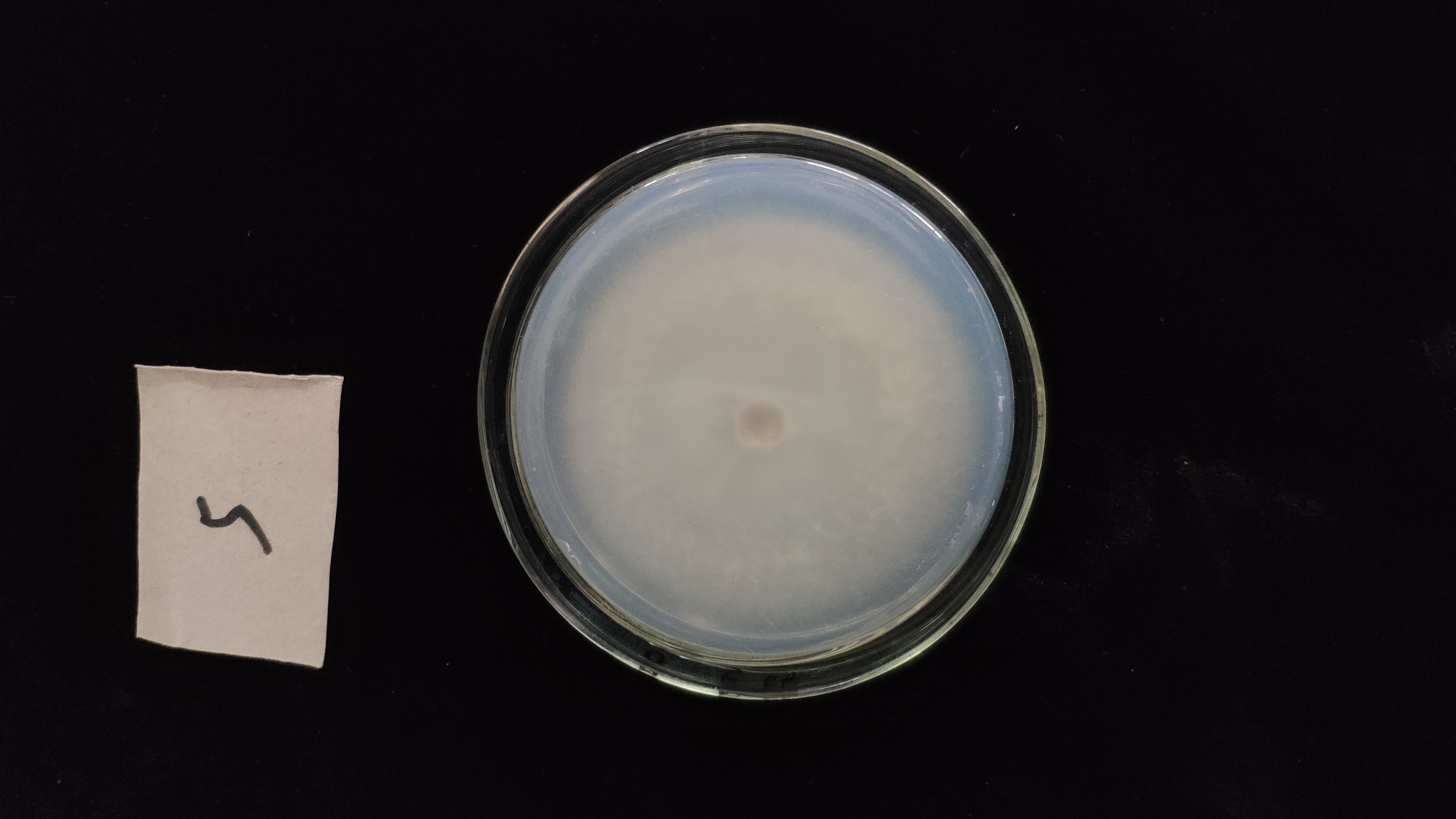

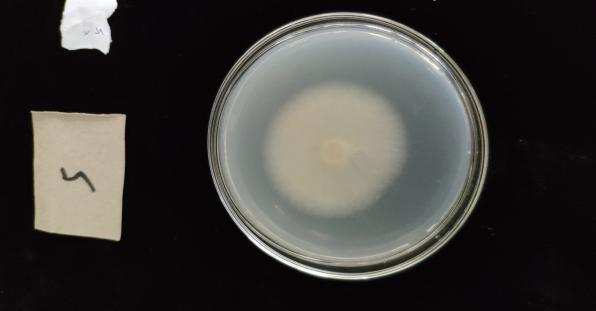

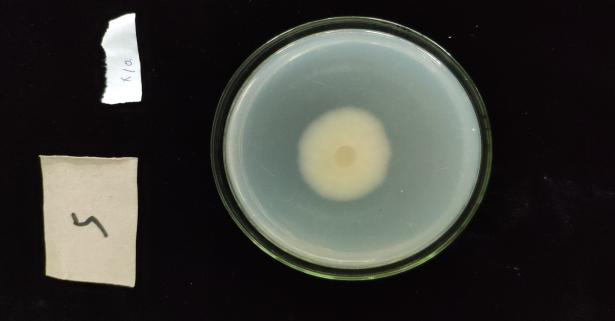

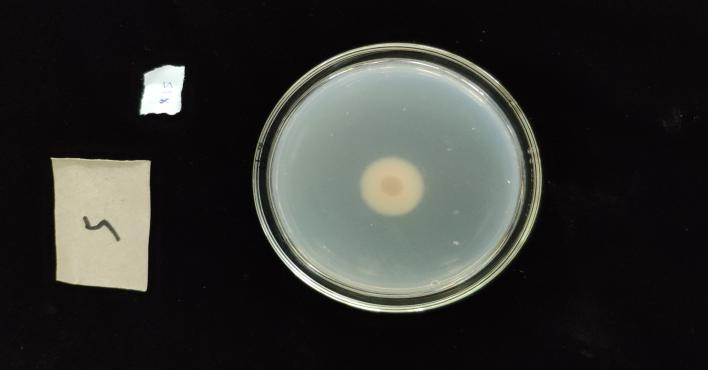

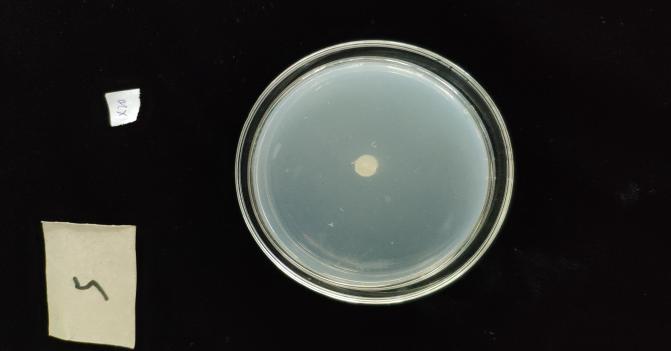

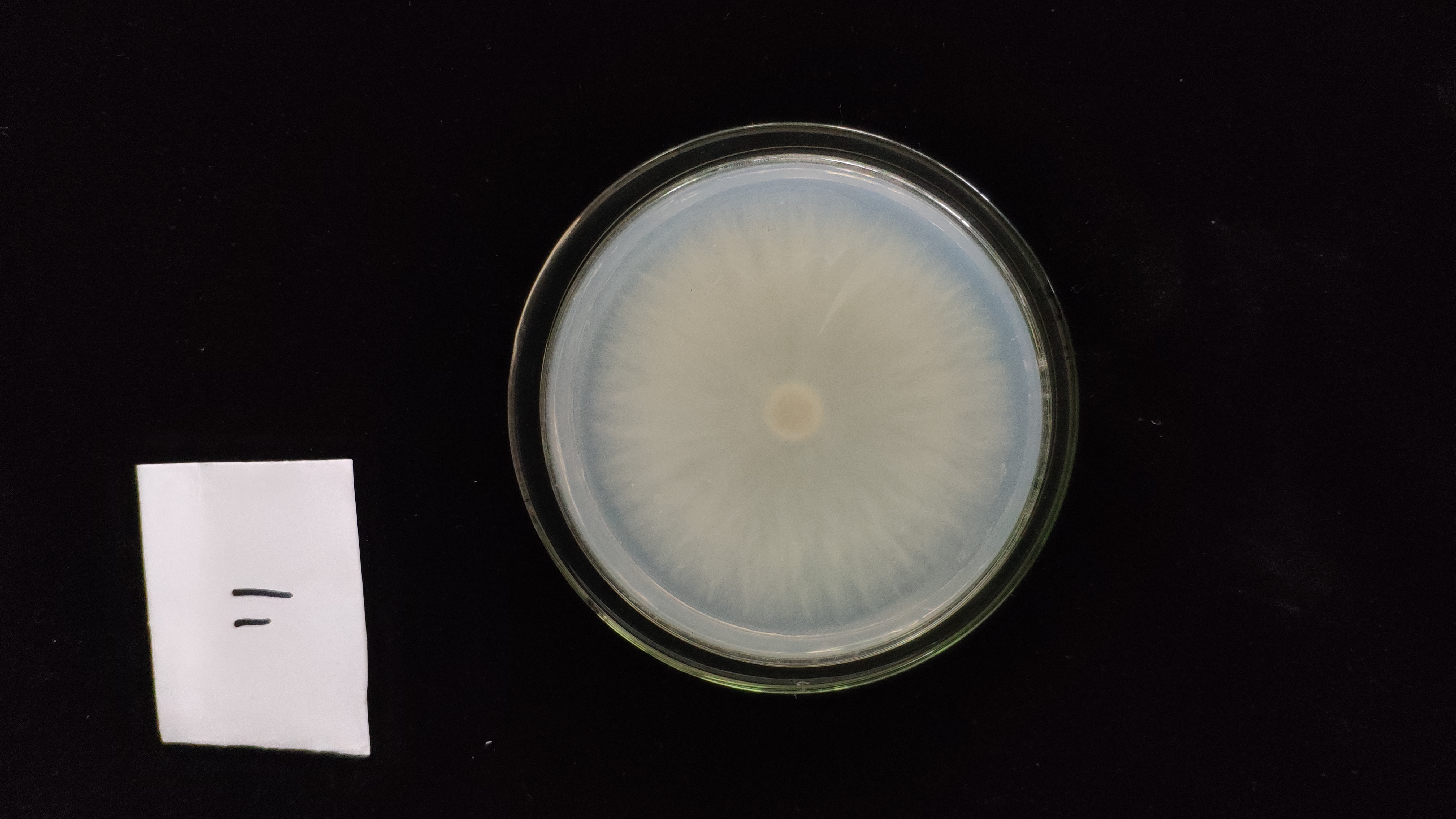

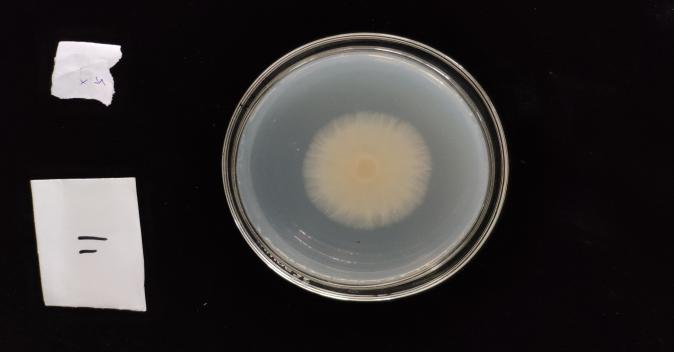

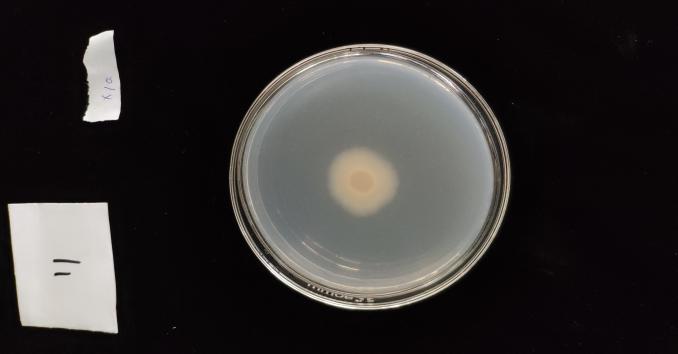

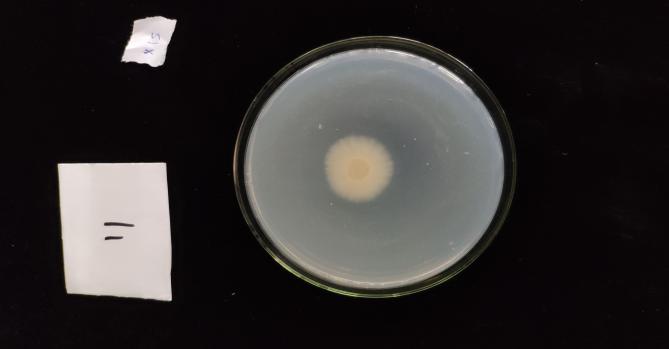

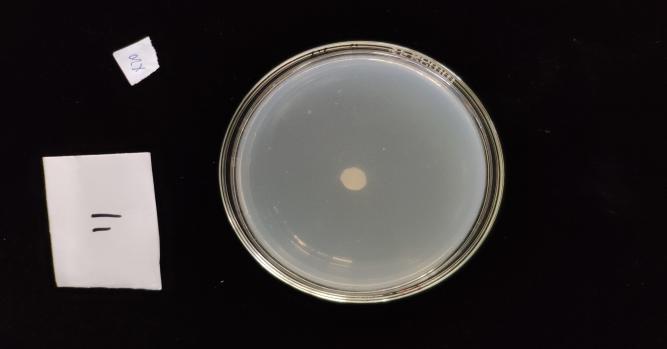


**a**

**b**

**c**

**d**

**e**

**k**

**l**

**m**

**n**

**o**

**f**

**g**

**h**

**i**

**j**

**p**

**q**

**r**

**s**

**t**

Fig.S6 Inhibition of pathogenic fungi by different concentrations of Paeonol and ethyl palmitate

(a-j: Paeonol; a-e: Y-5, f~j: Y-11; a, f: CK; b, g: 0.05mg/mL; c, h: 0.2mg/mL; d, i: 0.5mg/mL; e, j: 1.0mg/mL. k-t: Apocynin; k~o: Y-5, p~t: Y-11; k, p: CK; l, q: 0.5mg/mL; m, r: 1.0mg/mL; n, s: 1.5mg/mL; o, t: 2.0mg/mL.)


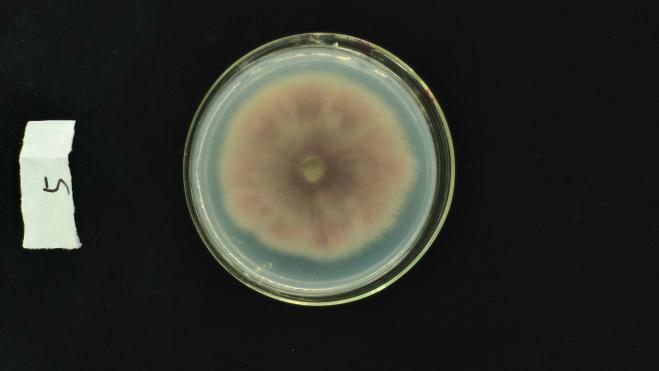

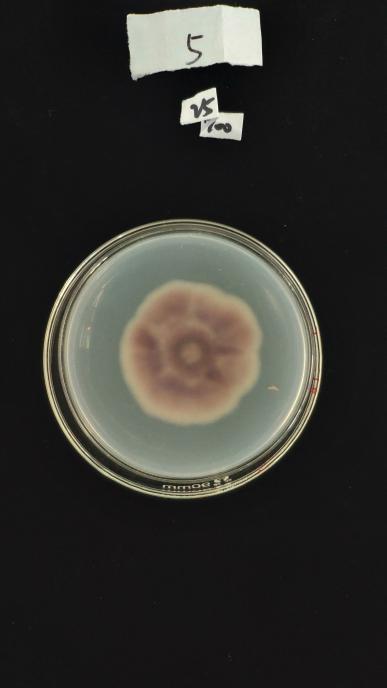

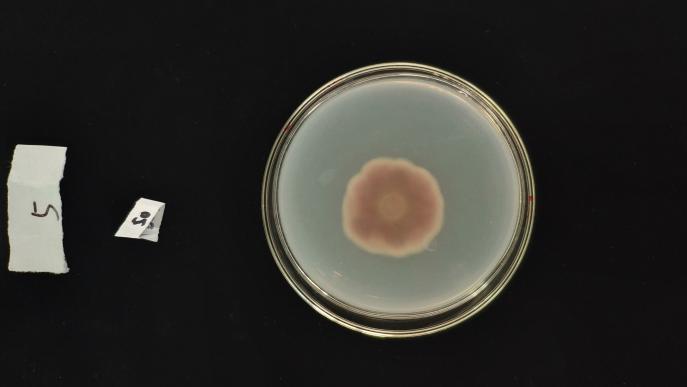

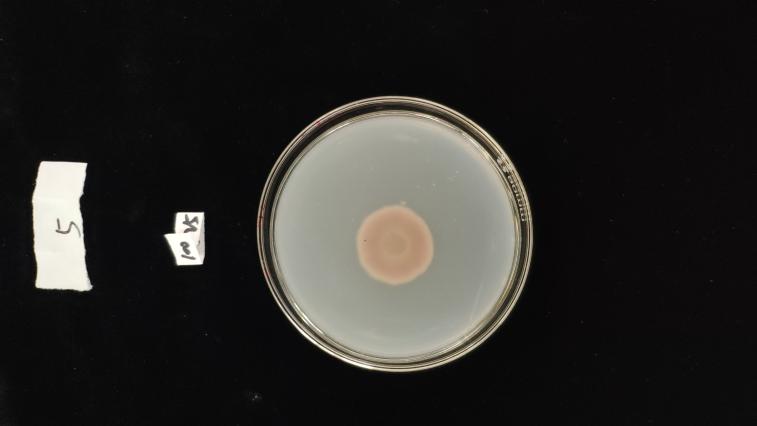

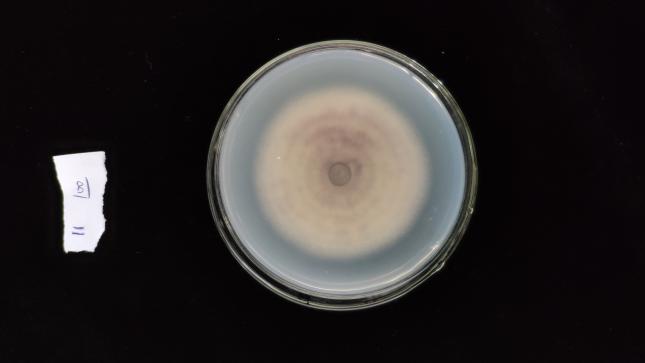

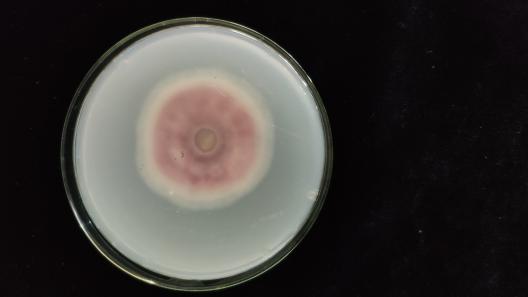

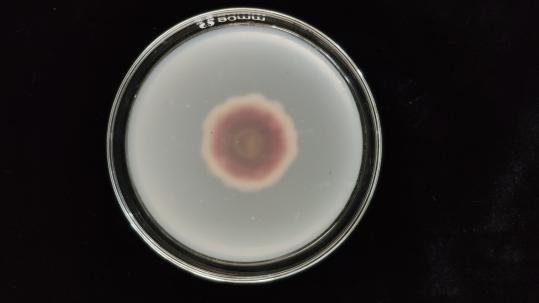

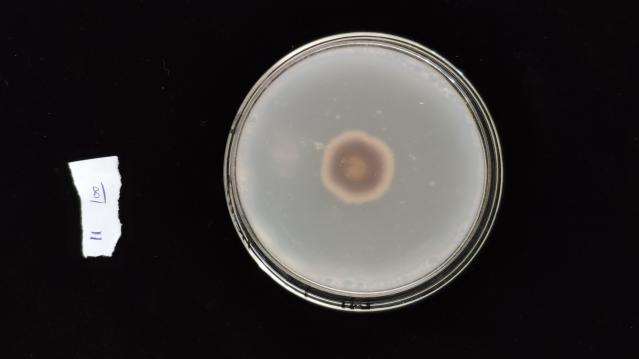


**a**

**b**

**c**

**d**

**e**

**f**

**g**

**h**

Fig.S7 Inhibitory effects of different concentrations of exogenous oxalic acid on pathogenic fungi

1. d:Y-5, e-h: Y-11; a,e: 0 mmol/L; b,f: 25 mmol/L; c,g: 50 mmol/L; d,h: 100 mmol/L)


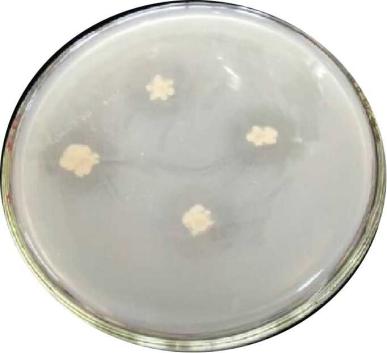


Fig.S8 Qualitative Study on Chitinase Production by C3

Table S1 Main active ingredients in C3 fermentation supernatant

| NO. | Compounds | CAS | RT | RI | Area % | Type |
| --- | --- | --- | --- | --- | --- | --- |
| P1 | ethyl palmitate | 628-97-7 | 26.99 | 1978 | 30.51 | Esters |
| P2 | Heptadecanoic acid, 15-methyl-, ethyl ester | 57274-46-1 | 30.61 | - | 9.58 | Esters |
| P3 | Ethyl benzoate | 93-89-0 | 8.59 | 1153 | 6.59 | Ketones |
| P4 | Paeonal | 552-41-0 | 15.12 | 1433 | 5.36 | Esters |
| P5 | Ethyl nonanoate | 123-29-5 | 11.45 | 1319 | 4.62 | Esters |
| P6 | 1-(2,4-Diethoxy-phenyl)-ethanone | 22924-18-1 | 16.97 | - | 3.16 | Ketones |
| P7 | Myristic acid, ethyl ester | 124-06-1 | 22.96 | 1795 | 3.15 | Esters |
| P8 | Elaidic acid, ethyl ester | 6114-18-7 | 30.26 | 2174 | 3.01 | Esters |
